# *Smyca*-FOXM1 ribonucleoprotein complex promotes homologous recombination and tumor immune evasion to define a therapeutic target of triple-negative breast cancer

**DOI:** 10.1186/s12929-026-01279-2

**Published:** 2026-08-11

**Authors:** Han-Hsiun Chen, Keng-Hao Chang, Sin-Rong Lee, Chih-Hao Chiu, Bing-Yu Yao, Tera Carissa, Shih-Duo Hsu Hung, Wen-Ling Kuo, Lily Hui-Ching Wang, Chia-Wei Li, Che-Ming Hu, Hsin-Yi Chen, Ruey-Hwa Chen

**Affiliations:** 1 Institute of Biological Chemistry, Academia Sinica, Taipei, 115 Taiwan; 2 Institute of Biochemical Sciences, College of Life Science, National Taiwan University, Taipei, 106 Taiwan; 3 Institue of Molecular Medicine, College of Medicine, National Taiwan University, Taipei, 100 Taiwan; 4 Institute of Biomedical Sciences, Academia Sinica, Taipei, 115 Taiwan; 5 School of Life Sciences, Department of Bioinformatics, Indonesia International Institute for Life Sciences, Jakarta, 13210 Indonesia; 6 Graduate Institute of Cancer Biology and Drug Discovery, College of Medical Science and Technology, Taipei Medical University, Taipei, 110 Taiwan; 7 Division of General and Breast Surgery, Department of Surgery, Chang Gung Memorial Hospital, Linkou, 244 Taoyuan Taiwan; 8 Institute of Molecular and Cellular Biology, National Tsing Hua University, Hsinchu, 300 Taiwan; 9 School of Medicine, National Tsing Hua University, Hsinchu, 300 Taiwan; 10 Ph.D. Program for Cancer Molecular Biology and Drug Discovery, College of Medical Science and Technology, Taipei Medical University, Taipei, 110 Taiwan

**Keywords:** Triple-negative breast cancer, Ribonucleoprotein complex, Long non-coding RNA, FOXM1, Homologous recombination, CGAS/STING pathway, Anti-tumor immunity

## Abstract

**Background:**

Triple-negative breast cancer (TNBC) is the most aggressive subtype of breast cancer with limited treatment options. Although PARP inhibitor (PARPi) offers great promise in treating TNBC with deficiency in homologous recombination (HR), most TNBC patients are HR-proficient. Furthermore, acquired resistance to PARPi remains as a challenge. Thus, there is an unmet need to identify new therapeutic target for developing advanced TNBC treatment strategy.

**Methods:**

I-SceI reporter assay and alkaline comet assay were used to analyze the role of *Smyca* in HR repair. Ingenuity pathway analysis was used to identify upstream regulators of *Smyca*-regulated transcriptome. RNA immunoprecipitation and RNA pull down were used to examine *Smyca*-FOXM1 interaction. Chromatin immunoprecipitation followed by sequencing was performed to identify FOXM1 target genes that are regulated by *Smyca*. Chromatin isolation by RNA purification was used to determine *Smyca* loading onto the promoters of FOXM1 target genes. Patient-derived organoid and xenograft mouse models were performed to evaluate the effect of *Smyca* on chemoresistance. Nanoparticle-assisted gapmer antisense oligonucleotides delivery was used to target *Smyca *in vivo. Co-culture of CD3 + T cells with TNBC cells and syngeneic mouse model were used to examine the effect of *Smyca*-FOXM1 targeting on anti-tumor immunity.

**Results:**

The long non-coding RNA *Smyca* is highly expressed in TNBC. We show that *Smyca* is induced by genotoxic agents to enhance HR repair. Mechanistically, *Smyca* binds FOXM1 and promotes the recruitment of FOXM1 to the promoters of a set of HR and nucleotide metabolism genes, thereby promoting their expression. *Smyca* ablation induces BRCAness in HR-proficient TNBC, thereby sensitizing these tumors to platinum or PARPi. Furthermore, targeting *Smyca*-FOXM1 complex in combination with platinum or PARPi activates cGAS/STING pathway and tumor immunogenicity to enhance anti-tumor immune surveillance. Clinically, *Smyca* expression in breast cancer patients correlates positively with therapy resistance and negatively with HR deficiency, interferon signature, and infiltration of anti-tumor immune cells.

**Conclusions:**

Our study identifies an unprecedented role of *Smyca* in HR repair to promote TNBC survival and immune evasion in response to therapy and suggests *Smyca* as a potential target for sensitizing TNBC to chemotherapy, PARPi, or immunotherapy.

**Supplementary Information:**

The online version contains supplementary material available at 10.1186/s12929-026-01279-2.

## Background

Triple-negative breast cancer (TNBC) is the most aggressive subtype of breast cancer with the worst prognosis. Because of lacking molecular markers, chemotherapy remains as the standard therapy for TNBC [1,2]. The discovery of synthetic lethality of PARP inhibitor (PARPi) in *BRCA1/2* mutant tumor cells offers a promising new approach for treating this type of TNBC patients [3,4]. The term of BRCAness is used to define phenotypes derived from homologous recombination (HR) deficiency upon *BRCA1/2* mutations, such as increased genome instability and sensitization to DNA damage agents such as platinum, topoisomerase I inhibitors, and PARPi [5]. More recently, the concept of BRCAness has been extended to the deficiencies in other essential HR genes and DNA damage response genes [6]. However, most TNBC patients (~ 65%) are HR-proficient and therefore cannot benefit from such treatment [7]. Even for HR-deficient patients, acquired resistance to PARPi remains as a clinically significant challenge [8,9]. Among the resistance mechanisms, restoration of HR activity/capacity by reactivation of HR genes or suppression of non-homologous end joining (NHEJ) activity are common [10–15]. Thus, a strategy to target the HR pathway would not only sensitize more TNBC patients to PARPi but also revert treatment resistance or reduce its occurrence in certain patients.

Among the various targets that sensitize or re-sensitize TNBC to PARPi, FOXM1 is likely a prominent candidate. FOXM1 is a forkhead-family transcription factor expressed at high levels in breast cancer, especially in TNBC [16,17]. A recent study revealed that FOXM1 undergoes liquid–liquid phase separation (LLPS) through an intrinsically disordered region and this LLPS capacity is enhanced in the presence of DNA with FOXM1-binding consensus sequences. Consequently, the formation of FOXM1 condensates allows the compartmentalization of active transcription machinery towards a set of oncogenes [18]. Indeed, FOXM1 elicits multiple tumor-promoting effects, such as invasion, metastasis, angiogenesis, epithelial-mesenchymal transition (EMT), cell cycle progression, apoptosis inhibition, and DNA repair [19,20]. FOXM1 promotes DNA repair by transcriptional activation of a set of genes involving in DNA damage sensing, signaling, and repair [21]. In the HR pathway, FOXM1 upregulates NBS1, thereby stabilizing MRN complex for damage recognition [22]. FOXM1 also enhances the transcription of EXO1 for DNA end resection [23], BRCA2, BRIP1, and RAD51 for strand invasion and displacement [24,25], and POLE2 and RFC2 for gap-filling [23]. Consistent with the key role of FOXM1 in multiple steps of the HR pathway, targeting FOXM1 by a small molecular inhibitor sensitizes HR-proficient TNBC cells to PARPi [17]. Furthermore, induction of FOXM1 after PARPi treatment was found in certain tumors to result in an adaptive response and FOXM1 ablation induces BRCAness to enhance PARPi sensitivity in this circumstance [26].

Deficiency in the HR-mediated repair of DNA double strand breaks (DSBs) is known to induce chromosome instability and micronuclei formation, which in turn activates the cGAS/STING innate immune pathway [27,28]. Consistently, in the BRCA-deficient tumor models, PARPi induces the activation of cGAS/STING pathway to enhance anti-tumor immunity and cytotoxic T cell infiltration [29,30]. Clinically, RNA-seq analysis of paired TNBC biopsy samples before and after PARPi treatment revealed the post-treatment induction of STING expression and type I and type II interferon (IFN) signaling [31]. In addition, PARPi upregulates PD-L1 expression through STING-dependent and independent mechanisms [32,33]. Thus, besides eliciting a cytotoxic effect directly on tumor cells, PARPi reshapes an anti-tumor immune microenvironment by IFN induction to assist the immune cells-mediated killing of HR-deficient TNBC and to sensitize TNBC cells to immune checkpoint blockades by inducing PD-L1 expression.

RNA-targeted therapeutics have recently received growing attentions by their potentials for treating and preventing human diseases [34]. In addition to mRNAs, long non-coding RNAs (lncRNAs) have emerged as a class of therapeutic targets. Compared to mRNAs, lncRNAs exhibit more tissue- and cell type-specific expression patterns and therefore could be ideal targets for personalized therapy [35]. In principle, lncRNAs that promote HR could be exploited as targets for enhancing tumor sensitivity to radiotherapy and chemotherapy. To date, several HR-promoting lncRNAs have been identified and their targets include RAD51, BRCA1, ATM, or ATR [36]. However, lncRNAs that promote multiple steps of the HR pathway remain to be discovered.

*Smyca* (Smad/Myc coactivator; also known as LOC284454) is a chromatin-enriched, 1.7 kb lncRNA. *Smyca* is highly expressed in TNBC and its high expression correlates with poor prognosis of many cancer types, including breast cancer [37–39]. Functionally, *Smyca* enhances Smad3/Smad4 complex formation and its recruitment to the promoters of target genes. Furthermore, *Smyca* binds c-Myc to promote c-Myc/Max complex recruitment to its target genes. Through activating both TGF-β/Smad pathway and c-Myc pathway, *Smyca* not only neutralizes the cytostatic effect of TGF-β by stimulating c-Myc-mediated cell proliferation, but promotes multiple malignant features elicited by the two pathways, including EMT, metastasis, stemness, metabolic rewiring, and chemoresistance [37]. In this study, we show that *Smyca* stimulates HR repair by enhancing the recruitment of FOXM1 to the promoters of a set of HR and nucleotide metabolism genes, and thus *Smyca* targeting in HR-proficient TNBC cells induces BRCAness phenotypes. Targeting *Smyca*/FOXM1 axis further activates cGAS/STING pathway and tumor immunogenicity-related gene expression to reshape an anti-tumor immune microenvironment.

## Methods

### Cell culture and transfection

Mouse TNBC cell line 4T1 (a gift from Yu-Ru Lee, Academia Sinica, Taipei, Taiwan) was cultured in Dulbecco’s Modified Eagle’s medium (DMEM)-F12 supplemented with 10% fetal bovine serum (FBS) and 1% penicillin/streptomycin (P/S; Gibco). HR and NHEJ reporter-expressing cells DR-GFP U2OS and EF5-GFP U2OS, respectively, and human TNBC cell line MDA-MB-436 were obtained from Peter Chi (National Taiwan University, Taipei, Taiwan). MDA-MB-231, MDA-MB-468, Hs578T, BT549 (purchased from BCRC, Taiwan), DR-GFP U2OS and EF5-GFP U2OS cells were cultured in DMEM supplemented with 10% FBS and 1% P/S. LM6 cells were cultured in DMEM supplemented with 10% FBS, 1% P/S, 1% sodium pyruvate, and 1% L-glutamine. MDA-MB-231 and Hs578T cells stably expressing *Smyca* shRNAs were described previously [37]. TALL-104 T lymphoblast cell line (a gift from Yu-Ching Lee, Taipei Medical University, Taipei, Taiwan) was cultured in RPMI-1640 supplemented with 20% FBS and 1% P/S. When it is needed, nucleosides (adenosine, guanosine, thymidine, and cytidine; 12.5 µM each) were supplemented to the culture medium. Transfection of plasmids and gapmer antisense oligonucleotides (ASOs) was performed with TransIT-X2 delivery system (Mirus) according to the manufacturer’s instructions. The sequences of gapmer ASOs are listed in Table S4, Supporting Information.

### Establishment of primary TNBC cells from a patient specimen

KWL-1001 cells were derived from tumor biopsy of a 41-year-old woman with right metaplastic carcinoma stage IV with the approval of the Institutional Review Board of the Chang Gung Medical Foundation (IRB No: 202002022A3). Tissues were washed three times with phosphate-buffered saline (PBS) containing 100 U/ml P/S, and cut up with scissors into small pieces in sterile DMEM/F-12 medium containing 100 U/ml P/S under sterile conditions. Single-cell suspension was prepared with the RWD tissue dissociator (DSC-410) according to the manufacturer’s instructions. The resulting cell suspension was filtered through a 45-µm nylon membrane filter and washed three times with PBS. The cells were pelleted down by centrifugation at 100 × *g* for 5 min, washed with PBS, seeded onto a type I collagen (Corning)-coated petri dish, and cultured for 2 weeks with daily medium changes in DMEM/F12 supplemented with 15% FBS, 20 ng/ml epidermal growth factor, ITS Premix (5 μg/ml insulin, 5 μg/ml human transferrin, and 5 ng/ml selenic acid; BD), and 0.25 μm dexamethasone (Sigma). Then, the cells were routinely cultured in the same medium and passaged every 4–5 days.

### Isolation, culturing and activation of human CD3^+^ T cells

Human peripheral blood mononuclear cells (PBMCs; PromoCell, Cat. No. C-12907) were resuspended in MACS buffer (PBS, pH 7.2, 0.5% BSA, and 2 mM EDTA) at a density of 1 × 10^7^ cells per 80 μl. CD3 MicroBeads (Miltenyi Biotec) were added according to the manufacturer’s instructions, and the cell suspension was incubated at 4 °C for 15 min. Cells were then washed with 2 ml MACS buffer and centrifuged at 300 × *g* for 5 min. The pellet was resuspended in 500 μl MACS buffer and subjected to magnetic separation using MS columns and a µMACS™ Separator (Miltenyi Biotec). CD3⁺ T cells were eluted with 2 ml RPMI-1640 complete medium. After centrifugation, the cell pellet was supplemented in TexMACS™ medium (Miltenyi Biotec) containing 10% FBS, 1% P/S, and 100 IU/ml recombinant human interleukin-2 (IL-2) (R&D Systems). The isolated CD3⁺ T cells were then activated by TransAct™ (Miltenyi Biotec) and 200 IU/ml recombinant human IL-2 for 3 days.

### CRISPR knockout (KO)

For generating FOXM1 KO or BRCA1 KO cells, lentivirus-based all-in-one CRISPR/Cas9 expression constructs for targeting FOXM1 or BRCA1 were obtained from RNA Technology Platforms and Gene Manipulation Core Facility (Taipei, Taiwan) or constructed in-house, respectively, and then transfected to MDA-MB-231 cells. The transfected cells were selected by puromycin and then subjected to limiting dilution to establish single cell-derived colonies. The KO efficiency was determined by Western blot. The sequences of sgRNAs are listed in Table S4, Supporting Information.

### Plasmids

Plasmid encoding Myc-Flag-STING was described previously [40]. Plasmid pCBASceI was provided by Peter Chi (National Taiwan University, Taipei, Taiwan). To generate the FOXM1 reporter construct, a pair of primers containing 6 × FOXM1-binding element (the FKH motif) was annealed and inserted into the pGL3 vector. The deletion mutants of *Smyca* were described previously [37].The FOXM1 dNRD, and dTAD mutants were generated by in vitro mutagenesis, whereas the FOXM1 dIDR-TAD mutant was made by PCR-based strategy. The primer sequences are listed in Table S5, Supporting Information.

### Reagents

Olaparib, G140, and FDI-6 were from MedChemExpress, whereas cisplatin, ATM inhibitor KU-55399, ATR inhibitor VE-821, DNA-PK inhibitor NU7441, and TGF-β RI inhibitor SB431542 were obtained from Sigma-Aldrich. STAT3 inhibitor LLL12 and AP-1 inhibitor T-5224 were purchased from MedChemExpress.

### HR and NHEJ reporter assays

For measuring HR and NHEJ activities, DR-GFP U2OS and EJ5-GFP U2OS cells were co-transfected with the I-SceI-expressing plasmid pCBASceI together with *Smyca* ASO or control ASO. On the next day, GFP-positive cells were analyzed using a Beckman Coulter CytoFlex Flow Cytometer with the CytoExpert v2.6 software.

### Alkaline comet assay

Comet assay was performed with the Comet Assay Kit (R&D Systems). Briefly, cells treated with various agents were harvested and re-suspended in PBS at a density of 2 × 10^5^ cells/ml, and then mixed with LMAgarose at a ratio of 1:9. The mixed solution was spread on the CometSlide^™^ and cooled at 4 °C for 30 min. The slides were incubated with Comet lysis solution at 4 °C for 1 h and then with unwinding solution (0.2 m NaOH and 1 mm EDTA) for 20 min at room temperature. The slides were electrophoresed in the electrophoresis solution at 4 °C and 20 V for 30 min, washed twice with deionized water for 5 min each, and then with 70% ethanol for 5 min. The slides were dried at 37 °C for 15 min and then stained with 100 μl of SYBR^®^ Gold at room temperature for 30 min. The slides were examined by a Zeiss Axio Imager.A2 microscope equipped with a Zeiss Plan-Apochromat 10x/0.45 objective lens and images were collected by the Zeiss ZEN software (Black Edition). The comet tail moment (TM = % DNA in tail × tail length/100) was measured by OpenComet software (v1.3.1) according to the manufacturer’s instructions.

### Cell proliferation

To determine the proliferation ability, cells were seeded on 96-well plates at a density of 2000 cells/well. After overnight culture, cells were labeled with 10 μm BrdU for 2 h, followed by fixation. Then, the BrdU incorporation was determined by the BrdU Cell Proliferation Assay Kit (Merck Millipore) according to the manufacturer's instructions.

### Luciferase reporter assay

Cells were transfected with pGL3-FOXM1 or pLuc-IFNβ Firefly luciferase plasmid together with pRK5F-based Renilla luciferase plasmid. After 24 h, cells were treated with FDI-6 or untreated for another 24 h. The Firefly and Renilla luciferase activities were detected using the Dual-Glo Luciferase Reporter Assay System (Promega), and then the Firefly luciferase activity was normalized to the Renilla luciferase activity.

### RNA pull-down analysis

Biotin-labeled *Smyca* RNA and its deletion mutants were synthesized via in vitro transcription using the AmpliScribe T7-Flash Biotin-RNA Transcription Kit (Epicentre Biotechnologies) and subsequently purified with the NucleoSpin RNA Kit (Macherey–Nagel). To ensure proper secondary structure formation, 30 pmol of biotinylated RNA was heated to 90 °C in RNA structure buffer (10 mM Tris, pH 7.0, 100 mM KCl, and 10 mM MgCl₂) for 2 min, chilled on ice for 2 min, and then incubated at room temperature for 20 min. The folded RNAs were incubated with recombinant GST-FOXM1 (Abnova) for 1 h at room temperature and then with Streptavidin magnetic beads (GE Healthcare) at room temperature for 1 h. After washes, the pull down complexes were analyzed by Western blot. Alternatively, the folded *Smyca* RNA was incubated with Flag-FOXM1 or its truncation mutants bound on M2 beads for 1 h. After washes, the pull down RNAs were extracted by TRIZOL and analyzed by RT-qPCR.

### Reverse transcription-quantitative PCR (RT-qPCR)

Total cellular RNAs were extracted using Trizol reagent (Invitrogen). cDNAs were synthesized from 2 μg RNA using iScript cDNA Synthesis Kit (Bio-Rad). Real-time qPCR was performed using LightCycler^®^ 480 SYBR Green I Master Kit (Roche) and amplified by Roche LightCycler 480 system. The PCR primer sequences are listed in Table S5, Supporting Information.

### Rolling-circle amplification

Deoxyribonucleoside triphosphates (dNTPs) synthesis levels in cells were determined by rolling-circle amplification as described [41]. Briefly, 1 × 10^6^ cells were lysed in 100 µl of 60% methanol before heating in a 100 °C heater for 10 min. Next, 100 µl of chloroform was added and mixed by vortex. The solution was centrifuged to separate the organic and aqueous layers. 50 µl of the aqueous layer was collected as the resource for dNTPs quantification. For rolling-circle amplification, phi29 DNA polymerase (New England Biolabs) was used. Each reaction was carried out at 37 °C for 50 min in a 10 µl of mixture containing 10^–17^ M circular DNA (template), 10^–12^ M primer, and three excessive dNTP (20 µM) with one limiting dNTP extracted from cells. After terminating the reaction at 65 °C for 10 min, the amplification products were diluted 5 folds and the level of each dNTP was quantified by qPCR. Ct value acquired from the blank group of reaction was used for normalization. The sequences of PCR primers are listed in Table S5, Supporting Information.

### Western blot and immunoprecipitation

Cells were lysed with RIPA buffer containing 50 mM Tris (pH 7.5), 150 mM NaCl, 1% NP-40, 0.1% SDS, 0.5% deoxycholic acid, 1 mM DTT, 1 mM phenylmethylsulphonyl fluoride (PMSF), 1 mM sodium vanadate, 1 μg/ml aprotinin, 1 μg/ml leupeptin, 2 mM sodium pyrophosphate and 200 μM NaF. Proteins were quantified by Bradford reagent. Western blot and immunoprecipitation using cell lysates with equal amounts of proteins were performed as previously described [40]. The antibodies used are listed in Table S6, Supporting Information.

### RNA immunoprecipitation (RIP)

Cells were lysed with polysome lysis buffer containing 15 mM Tris (pH 7.5), 300 mM NaCl, 1% Triton X-100, 1 mM DTT, 1 mM PMSF, 1 μg/ml aprotinin, 1 μg/ml leupeptin, 1 mM sodium vanadate, 4 mM sodium pyrophosphate, 20 mM NaF, and 100 U/ml SUPERaseIN. The lysates were pre-cleared by incubating with Protein-A magnetic beads (Millipore) at 4 °C for 60 min. Next, 4 μg of antibody was added to the pre-cleared lysates, and the immunoprecipitated protein-RNA complex was captured using Protein-A magnetic beads at 4 °C for 2 h. The immunoprecipitated proteins and co-precipitated RNAs were extracted from the beads using sample buffer and TRIZOL reagent, and analyzed by RT-qPCR and Western blot, respectively.

### Chromatin isolation by RNA purification (ChIRP)

ChIRP was performed as described previously [37]. Briefly, cells were subjected to chromatin crosslinking with 1.25% glutaraldehyde for 15 min and then quenched with 125 mM glycine for 5 min. The cross-linked chromatins were sheared into 200–500 bp by sonication and hybridized with biotinylated tiling 20-mer ASO probes for targeting *Smyca* or negative control probes for targeting lacZ RNA. After streptavidin magnetic beads capturing and wash/elution steps, the ChIRP captured chromatins were treated with Protease/RNase and analyzed by qPCR. Data were normalized to DNA input and presented as a percentage of input. The sequences of ChIRP tilling probes and ChIRP-qPCR primers are listed in Table S4 and S5, Supporting Information, respectively.

### Chromatin immunoprecipitation (ChIP)

Chromatin isolation, sonication, and immunoprecipitation using anti-FOXM1 antibody were performed as described [37]. The FOXM1-bound DNA fragments were analyzed by qPCR using ChIP-qPCR primers listed in Table S5, Supporting Information. Alternatively, the FOXM1-bound DNA fragments were subjected to DNA library preparation using NEB Next ChIP-Seq Library Prep Reagent Set for Illumina (New England Biolabs), followed by qualification of the fragment length distributions and concentrations by KAPA Library Quantification Kits for Illumina (KAPA Biosystems), according to the manufacturer’s instructions. The ChIP-seq library was then subjected to high-throughput next-generation sequencing using HiSeq 2000 (Illumina) following the manufacturer’s protocols. Reference files of the human reference sequence assembly (NCBI Build 37/hg19, February 2009) and GTF annotation file were obtained from iGenomes (http://support.illumina.com/sequencing/sequencingsoftware/igenome.html). All ChIP-seq data sets were aligned using Bowtie. FOXM1-binding regions were identified using MACS software with a *P* value threshold of 1e-5, and peaks mapping within 5 kb (− 4.5 to + 0.5 kb) of a transcription start site were assigned to genes. A binding site was assigned to the nearest gene within 100 kb from a peak using CisGenome. The mapping of transcription factor binding sites to the specific genomic regions was calculated using CisGenome. The pathway enrichment analysis of *Smyca*-regulated FOXM1 targets was performed using Gene Ontology (GO) (http://www.geneontology.org/).

### Immunofluorescence

Cells seeded on coverslips were washed three times with PBS and fixed with 4% formaldehyde at room temperature for 20 min. After three washes with PBS, cells were permeabilized with ice-cold methanol for 15 min and washed three times with PBS. For RAD51 immunostaining, cells were pretreated with 1% Triton X-100 for 10 min before fixation. The fixed cells were permeabilized with 1% Triton X-100 for 10 min, and then blocked with PBS containing 1% BSA and 10% goat serum at room temperature for 1 h. The slides were incubated with primary antibody diluted in blocking buffer at 4 °C for overnight. Next, the slides were washed three times with PBS, rinsed once with blocking buffer, and then incubated with fluorescence-conjugated secondary antibody (Life Technologies) together with DAPI (1 μg/ml) (Sigma-Aldrich) at room temperature for 40 min. Cells were washed three times with PBS and mounted with a mounting medium (Dako). Confocal images were captured by Olympus FV3000 confocal microscope equipped with a 60x/1.40 oil PlanApo N Olympus objective lens. Images were collected by Olympus FV3000 FV31S-SW (v2.40) software. The primary and secondary antibodies used are listed in Table S6, Supporting Information.

### DNA fiber combing assay

Replication tract progression was examined by DNA fiber combing and was modified from previous methods [42,43]. Briefly, cells were seeded in 6-well plates at a density of 3 × 10^5^ cells/well. Cells were then labeled with 100 μM CldU for 30 min, washed three times with PBS, and exposed to 100 μM IdU together with 10 μM Olaparib for 30 min. Next, cells were collected by trypsinization, washed with cold PBS, and resuspended in PBS containing 0.05% trypsin. The cell suspension was mixed with molten 1.2% low-melting-point agarose and transferred into plug molds. Plugs were allowed to solidify at 4 °C for 2 h and then incubated overnight at 50 °C in ESP buffer (0.41 M EDTA, 0.9% sarkosyl, and 3.6 mg/mL proteinase K). Plugs were washed multiple times in TE buffer (10 mM Tris, pH 8.0, and 1 mM EDTA, pH 8.0), incubated in 0.5 M MES buffer (pH 5.5) at 68 °C for 20 min, and then held at 42 °C for 10 min. β-Agarase I (New England Biolabs) was added and incubation was continued for overnight at 42 °C to liberate genomic DNA. The released DNA was transferred to a reservoir containing 0.5 M MES buffer (pH 5.5) and stretched onto silanized coverslips by molecular combing. Both the combing apparatus and silanized coverslips were prepared and provided by Dr. Hungjiun Liaw (National Cheng Kung University, Tainan, Taiwan). Following combing, coverslips were dried at 65 °C for 2 h, denatured in 0.5 M NaOH/1 M NaCl for 10 min, washed three times in PBS, and dehydrated through 70% and 100% ethanol for 5 min each.

For tract detection, coverslips were blocked in filtered 5% BSA in PBS for 30 min at room temperature. CldU-labeled tracts were detected by incubating coverslips with rat anti-BrdU antibody for 90 min at room temperature, followed by PBS washes, fixation in 4% paraformaldehyde for 10 min, and staining with Alexa Fluor 594-conjugated goat anti-rat secondary antibody for 90 min at room temperature. IdU-labeled tracts were then detected by overnight incubation at room temperature with mouse anti-BrdU antibody, followed by Alexa Fluor 488-conjugated goat anti-mouse secondary antibody for 90 min. PBS washes were included between each staining step, and coverslips were finally mounted in antifade mounting medium. Images were acquired using Olympus FV4000-RS inverted confocal microscope equipped with an Olympus UPLXAPO 60 × oil immersion objective lens (NA 1.42). DNA fiber tract lengths were assessed by ImageJ software. The primary and secondary antibodies used are listed in Table S6, Supporting Information.

### Cell viability assay

All cells were seeded on 96-well plates at a density of 4000 cells/well, except for Hs578T (2000 cells/well) and KWL-1001 (8000 cells/well). Cells were then cultured in medium containing cisplatin for 3 days or olaparib for 5 days. Cell viability was determined by incubating with 0.4 mg/ml methyl thiazolyl diphenyl tetrazolium bromide (MTT, Sigma-Aldrich) for 2 h, followed by cell lysis with DMSO and absorbance measurement at 590 nm.

### ELISA assays

For measuring 2′3′-cGAMP, cells were lysed with NP40 lysis buffer containing 150 mM NaCl, 50 mM Tris–HCl (pH 7.5), 1% NP40, 1% sodium deoxycholate, 1 μg/ml aprotinin, 1 μg/ml leupeptin, and 1 mM PMSF. cGAMP levels were then measured by using the cGAMP ELISA Kit (Cayman) according to the manufacturer’s instructions. For detecting IFNβ concentrations, culture medium was collected and measured using the human IFNβ ELISA Kit (R&D System) according to the manufacturer’s instructions.

### Transwell migration assay

Activated human CD3^+^ T cells were live-stained with Hoechst 33342 (Sigma) and seeded on a Hanging Cell Culture Insert (Merck Millipore). MDA-MB-231 cells transfected with control ASO or *Smyca* ASO and treated with cisplatin or olaparib for 72 h were seeded on the lower chamber of 24-well plates. After 4 h of migration, medium in the lower chamber containing the migrated T cells was transferred to an 8 Well Glass Bottom µ-Slide (Ibidi). The fluorescence-labeled T cells were imaged by an Olympus scanR High-Content Screening Station equipped with Olympus LUCPlanFL N 20x/0.45 NA objective lens. The images were collected by Olympus cellSens software and quantified by ImageJ.

### T cell-mediated tumor killing assay

Conditioned medium (CM) was collected from MDA-MB-231 cells transfected with control ASO or *Smyca* ASO and treated with cisplatin or olaparib for 3 days. 1 × 10^4^ MDA-MB-231-luc cells [37] were seeded on 6-well plates. On the next day, the medium was replaced with the aforementioned CM containing 5 × 10^4^ TALL-104 cells. After two days of incubation, the remaining MDA-MB-231-luc cells were collected by trypsinization and their survival was measured by the Dual-Glo Luciferase Reporter Assay System (Promega) according to the manufacturer's instructions.

### Organoid survival assay

KWL-1001 cells transfected with control ASO or *Smyca* ASO were seeded on 96-well Clear Ultra Low Attachment Microplates (Corning^™^) at a density of 500 cells/well. On the next day, cells were treated with olaparib or cisplatin for 3 days and then the medium was changed to fresh culture medium to allow the growth of additional 8 days. The morphology of organoids was imaged using an Olympus CKX41 microscope equipped with Olympus LUCPlanFL N 4x/0.13 PhL objective lens. Cell viability was measured using the CellTiter-Glo^®^ 3D Cell Viability Assay kit (Promega) according to the manufacturer’s instructions.

### Immunohistochemistry (IHC)

Tumor sections were prepared by the Pathology Core Facility, Institute of Biomedical Sciences, Academia Sinica. IHC staining was conducted using the Novolink Max Polymer kit (Leica Biosystems) according to the manufacturer's protocol. In brief, slides were dewaxed with xylene and ethanol, and antigen retrieval was achieved by boiling in TRS buffer for 10 min. Slides were blocked with Protein Block for 10 min and incubated with primary antibody for overnight and then with polymer for 30 min. The peroxidase activity was visualized with diaminobenzidine tetrahydroxychloride (DAB). Finally, the sections were counterstained with hematoxylin (Sigma-Aldrich) and sealed with Sub-x mounting medium (Leica Biosystems). The IHC images were taken by a Zeiss Axio Imager A2 microscope equipped with Zeiss Plan-Apochromat 20x/0.75 NA infinity-corrected objective lens and collected by Zeiss ZEN software (Black Edition). Antibodies used are listed in Table S6, Supporting Information. Data were quantified by Image J.

### Preparation of lipid nanoparticles (LNPs)

Ionizable lipid [(6Z, 9Z, 28Z, 31Z)-heptatriaconta-6, 9, 28, 31-tetraen-19-yl] 4- (dimethylamino) butanoate (Dlin-MC3-DMA; #34365) and 1, 2-Dimyristoyl-rac-glycero-3-PE-methoxy-Polyethyleneglycol-2000 (DMPE-2kPEG; #34649) were obtained from Cayman Chemical. Cholesterol was sourced from Sigma-Aldrich and 1, 2-distearoyl-sn-glycero-3-phosphocholine (DSPC) was acquired from Avanti Polar Lipids, Inc. (Alabaster). LNPs were prepared under RNase-free conditions. Initially, a glass bottle and stir bar were cleaned with chloroform and dried using nitrogen gas. Next, 100 µg of *Smyca* gapmer ASO or control gapmer ASO was combined with 600 µl of 50 mm citrate buffer (Sigma-Aldrich). This solution was then mixed with 200 µl of an ethanolic lipid solution while stirring. The lipid mixture consisted of DLin-MC3-DMA, DSPC, cholesterol, and DMPE-2kPEG in a ratio of 50:10:38.5:1.5. Following a 4 h evaporation period in a vacuum chamber under gentle stirring, the solution was diluted with a 10% sucrose buffer (Fisher Scientific). The mixture was then concentrated using an Amicon^®^ centrifugal filter with a 100 kDa molecular-weight cut-off (Millipore Sigma) at 600 × *g* at 4 °C. The resulting LNPs were stored at − 80 °C.

### Animal studies

Mice were housed in a specific pathogen-free animal facility under temperature (22 ± 2°) and humidity-controlled (55 ± 5%) conditions with a 12 h light/12 h dark circadian cycle and access to food and water. For analyzing TNBC growth in xenograft model, 5 × 10^6^ MDA-MB-231 cell derivatives were orthotopically injected into the fat pad of 7-week-old female Nu/Nu (Bltw: NU-*Foxn1nu*) mice (Bio-LASCO Taiwan Co.). When tumors grow to ~ 100 mm^3^ in volume, cisplatin (2.5 mg/kg) or olaparib (125 mg/kg) diluted in 100 µl corn oil was intraperitoneally injected twice a week [44,45]. Alternatively, 2 × 10^6^ LM6 cells were orthotopically injected into the fat pad of 7-week-old female Nu/Nu mice. When tumors grow to ~ 100 mm^3^ in volume, LNPs containing *Smyca* ASO or control ASO were intratumorally injected once a week. Three days later, cisplatin (2.5 mg/kg) or olaparib (125 mg/kg) was intraperitoneally injected once a week. For analyzing tumor growth and tumor-infiltrating leucocytes (TILs) in syngeneic model, 1 × 10^6^ 4T1 cells were orthotopically injected into the fat pad of 7-week-old female BALB/c mice (Bio-LASCO Taiwan Co.). When tumors grow to ~ 200 mm^3^ in volume, FDI-6 (40 mg/kg) together with cisplatin (2.5 mg/kg) or olaparib (125 mg/kg) diluted in 100 µl corn oil was given via intraperitoneal injection twice a week. The sizes of tumors were measured twice a week, and their volumes were calculated using the equation mm^3^ = 1/2 × length (mm) × (width (mm))^2^. All mouse experiments were conducted with the approval from the Institutional Animal Care and Use Committee, Academia Sinica.

### Flow cytometry analysis of TILs

For analyzing TILs, 0.5 mg of tumors were minced and incubated with 10 ml digestion buffer containing HBSS (Sigma Aldrich), 5 mM CaCl_2_, 40 U/ml DNase I (Roche), 0.5 mg/ml type I collagenase (Gibco, ThermoFisher Scientific), 0.5 mg/ml type IV collagenase (Gibco, ThermoFisher Scientific), and 0.2 mg/ml hyaluronidase (MedChemExpress) for 30 min at 37 °C. The dissociated tumor cells were passed through a 70 μm cell strainer (BD Biosciences), incubated with ammonium chloride potassium (ACK) lysis buffer (MyBioSource), and counted for taking an equal number of live cells in each sample. After washing with PBS containing 2% inactivated FBS, cell suspensions were first stained with Fixable Viability Stain 780 (BD Biosciences) to exclude dead cells, blocked with anti-CD16/32 antibody (BioLegend), and stained with antibodies targeting immune cell surface markers for 60 min on ice. Afterwards, cells were fixed and permeabilized with Transcription Factor Buffer Set (BD Biosciences) for 50 min on ice and stained with antibodies targeting intracellular markers for 60 min on ice. Data were collected by a ThermoFisher Scientific Attune NxT Flow Cytometers and analyzed with FlowJo v10.8.1 software. Antibodies used are listed in Table S6, Supporting Information.

### Bioinformatics

The breast cancer (BRCA) mutational, transcriptomic, and clinical data were directly downloaded from TCGA using the TCGA biolinks package in R. The HR deficiency (HRD) score was defined as the mean value of 3 components of the HRD/genome scarring scores: HRD-LOH, LST, and NtAI per chromosome, which were generated by the TCGA Network Aneuploidy AWG using ABSOLUTE. To calculate the Tumor mutation burden (TMB) score, total number of mutations (somatic, coding, base substitution, and indel mutations) was divided by length of the region of targeted territory. IFN-stimulatory genes (ISG) signature scores were calculated with mean absolute deviation modified Z-score (ZMAD)-normalized mRNA expression data in TCGA. The score was defined as the mean ZMAD value of all Type I IFN signature genes in each sample. The levels of various TILs in the TCGA breast cancer cohort were revealed by the Immunedeconv R package. *Smyca* and FOXM1 gene expression were analyzed for the correlation with the abundance of various TILs. The correlations were visualized as a heatmap with numbers showing the purity-adjusted Spearman’s Rho and the *P* value. To discover the enriched sequence motifs around the ChIP peaks of 96 *Smyca*-regulated FOXM1 targets, ± 100 bp genomic sequence of *Smyca* regulated FOXM1 loaded peaks were extracted from ChIP-seq dataset and then subjected to MEME motif discovery analysis (https://meme-suite.org/meme/).

### Statistical analysis

All statistical analyses were performed by GraphPad Prism 8. Unpaired Student’s t-test was used for comparison between two groups, whereas one-way or two-way ANOVA was used for multigroup comparisons. Pearson’s correlation was used for data with normal distribution, such as gene expression data from patient cohorts. Spearman’s rank correlation was applied to analyzing TIL data based on the TIMER algorithm (https://compbio.cn/timer1/). *P* values are indicated in the figures and all data were presented as mean ± SD. For animal experiment, each mouse was considered as a biologically independent sample.

## Results

### *Smyca* is induced by DNA damage to promote HR repair

**Fig. 1:**
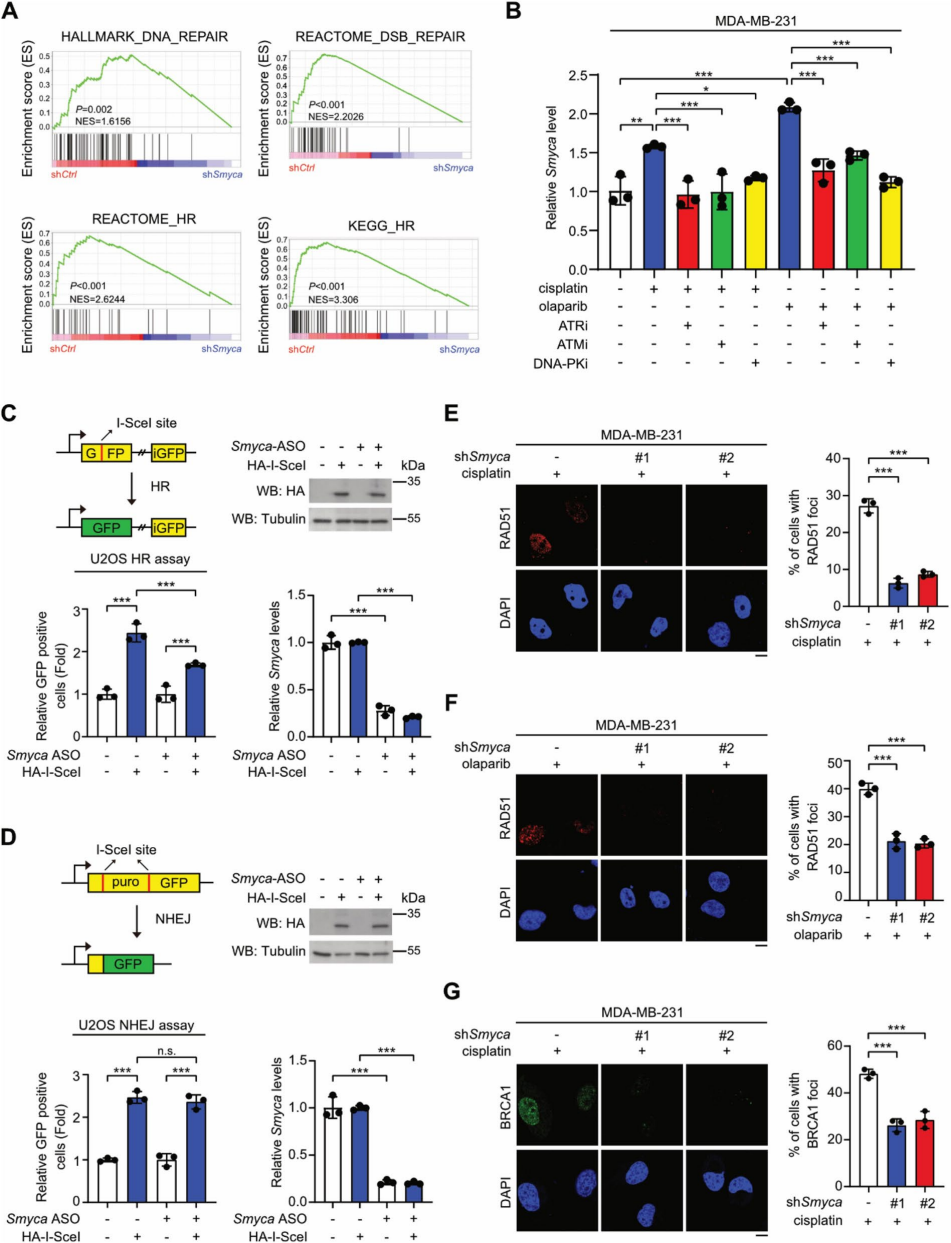
*Smyca* is induced by DNA damage to promote HR repair. **A** GSEA plots for the correlations of *Smyca*-regulated signature with DNA repair signatures. Enrichment score (ES) and normalized enrichment score (NES) are indicated. **B** RT-qPCR analysis of *Smyca* expression levels in MDA-MB-231 cells treated with 50 μM cisplatin or 10 μM olaparib for 72 h, together with or without 2.5 μM ATRi, 10 μM ATMi, or 5 μM DNA-PKi for 24 h. **C**, **D** Upper left panels: Schematic of the DR-GFP **C** and EJ5-GFP **D** reporter assays to assess the HR and NHEJ activities, respectively. Lower left panels: HR and NHEJ activities measured by flow cytometry analysis of GFP^+^ cells from DR-GFP U2OS and EJ5-GFP U2OS cells transfected with I-SceI expression plasmid and/or indicated ASOs. The equal expression of I-SceI and the knockdown efficiencies of *Smyca* ASO are shown on the upper right and lower right panels, respectively. Data in **B**, **C**, **D** are mean ± SD, n = 3. *P* values are determined by one-way ANOVA with Tukey’s post hoc test. **E**–**G** Representative confocal images of RAD51 **E**, **F** and BRCA2 **G** foci after 50 μM cisplatin **E**, **G** or 10 μM olaparib **F** treatment of indicated MDA-MB-231 cell derivatives for 4 h (for BRCA1 foci) or 2 h (for RAD51 foci). Bar, 10 μm. Data are mean ± SD, n = 3 and > 30 cells per group per experiment are analyzed. *P* values are determined by one-way ANOVA with Tukey’s post hoc test

In an attempt to explore unknown functions of *Smyca* in TNBC, we performed Gene Set Enrichment Assay (GSEA) with Hallmark pathway analysis of a previously reported *Smyca*-regulated transcriptome in MDA-MB-231 cells (GSE181028) [37]. Notably, “DNA repair” and “UV response up” were among the upregulated hallmarks, whereas “UV response down” was a downregulated hallmark (Figure S1A, Supporting Information). Furthermore, GSEA analysis showed that *Smyca-*governed transcriptome correlated positively with DNA repair, DSB repair, and HR signatures from different sources (Fig. 1A). In line with these bioinformatics findings, *Smyca* expression levels in TNBC cell lines MDA-MB-231 and Hs578T were increased by treating cells with platinum-based chemodrug, cisplatin or PARPi, olaparib (Fig. 1B and Figure S1B, Supporting Information). Inactivation of DNA damage-sensing kinase ATM, ATR, or DNA-PK impaired the induction of *Smyca* expression by DNA damage agents (Fig. 1B). Furthermore, by searching the University of California Santa Cruz (UCSC) Genome Browser (https://genome.ucsc.edu/), we revealed the loading of transcription factor AP-1 onto several 5′-regulatory regions of *Smyca* gene (Figure S1C, Supporting Information). Consistent with a role of AP-1 in DNA damage response (DDR) by acting downstream of DNA damage-sensing kinases [46], AP-1 inhibition by a small molecular inhibitor T-5224 abrogated genotoxic agent-induced *Smyca* upregulation (Figure S1D, Supporting Information). Collectively, these findings suggest that *Smyca* is upregulated by DDR/AP-1 axis to control DSB repair. We therefore evaluated the effects of *Smyca* on the two major DSB repair pathways, HR and NHEJ, using DR-GFP U2OS and EJ5-GFP U2OS reporter cells [47,48], respectively. In these assays, DSB in the reporters is generated by exogenous expression of I-SceI endonuclease and the subsequent HR or NHEJ repair leads to the restoration of GFP fluorescence (Fig. 1C, D, upper left panels). Importantly, depletion of *Smyca* by gapmer antisense oligonucleotide (ASO) diminished the HR repair activity without affecting the NHEJ repair activity (Fig. 1C, D). Furthermore, using previously established MDA-MB-231 and Hs578T cells stably expressing *Smyca* shRNAs [37], we found that *Smyca* depletion decreased RAD51 foci formation in response to cisplatin or olaparib treatment (Fig. 1E, F and Figure S1E, Supporting Information). *Smyca* knockdown in MDA-MB-231 and Hs578T cells also attenuated BRCA1 foci formation under cisplatin treatment (Fig. 1G and Figure S1F, Supporting Information). Thus, our data indicate that *Smyca* is induced by DNA damage to promote HR repair but not NHEJ repair.

**Fig. 2:**
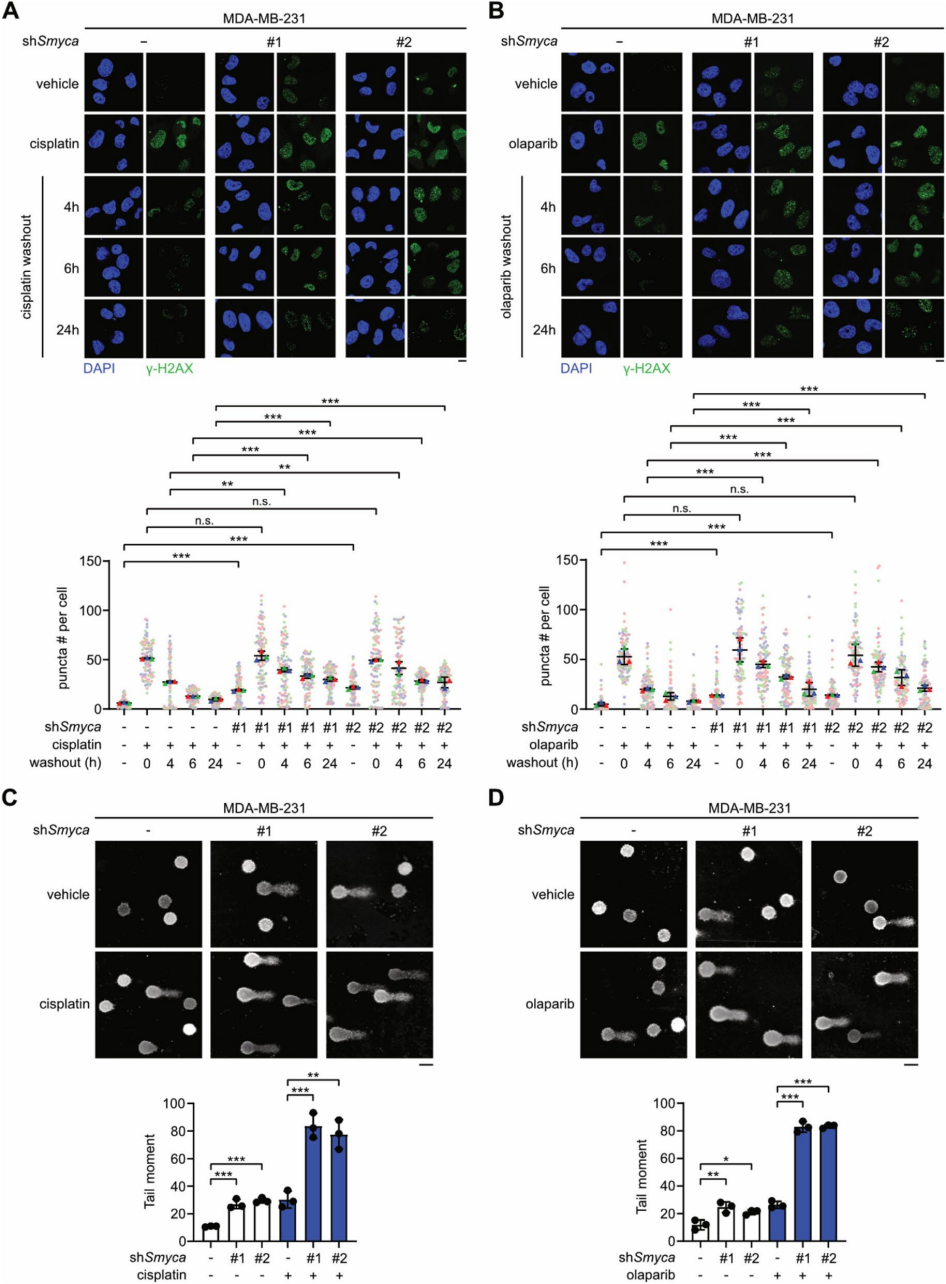
*Smyca* depletion compromises DNA DSB repair. **A**, **B** Representative confocal images and quantitative data for γH2AX foci in indicated MDA-MB-231 cell derivatives treated with 50 μM cisplatin **A** or 10 μM olaparib **B** for 4 h, followed by drug removal to allow DNA repair for indicated time periods. Bar, 10 μm. **C**, **D** Representative images and quantitative data for alkaline comet assay to detect the extent of DNA DSBs in indicated MDA-MB-231 cell derivatives treated with 50 μM cisplatin **C** or 10 μM olaparib **D** for 24 h. Bar, 50 μm. Data in all panels are mean ± SD, n = 3 and > 30 cells per group per experiment are analyzed. *P* values are determined by one-way ANOVA with Tukey’s post hoc test

Consistent with a key role of *Smyca* in HR repair, γH2AX staining showed that *Smyca*-depleted MDA-MB-231 and Hs578T cells exhibited increased DNA damage levels in the basal state and a delay in the repair of DNA damage after treatment with cisplatin or olaparib (Fig. 2A, B and Figure S2A, Supporting Information). *Smyca* depletion by ASO elicited a similar effect (Figure S2B, Supporting Information). Accordingly, alkaline comet assay revealed the increase of comet tail moments in *Smyca* knockdown MDA-MB-231 and Hs578T cells treated or untreated with cisplatin or olaparib (Fig. 2C, D and Figure S2C, Supporting Information). These findings substantiate the role of *Smyca* in DSB repair under basal and DNA damage conditions.

### *Smyca* binds FOXM1 and promotes FOXM1 recruitment to the promoters of HR genes for activating their transcription

**Fig. 3:**
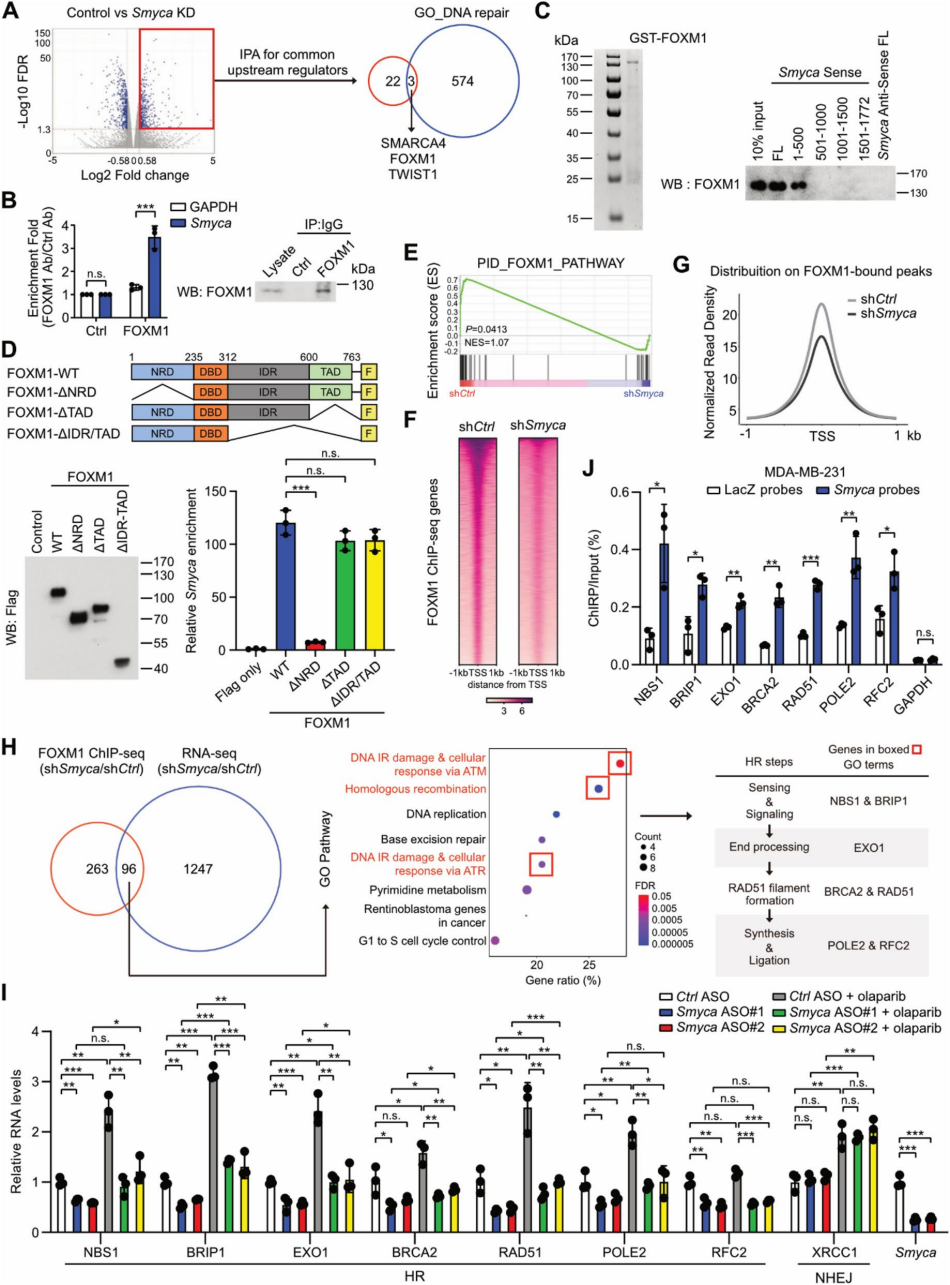
*Smyca* binds FOXM1 and promotes FOXM1-mediated transcription of HR genes. **A** The intersection of common upstream regulators of *Smyca*-upregulated genes revealed by IPA with the DNA repair genes in GO. The volcano plot was adapted from our previous study [37] and DEGs are marked by blue dots. **B** RIP analysis for the enrichment of *Smyca* in FOXM1 immunoprecipitates derived from MDA‑MB‑231 cells. Data are normalized with that from the control antibody and presented as mean ± SD, n = 3. *P* values are determined by unpaired t-test. The presence of FOXM1 in the immunoprecipitates is shown on the bottom. **C** In vitro binding assay using recombinant GST-FOXM1 and biotinylated *Smyca* full length or truncated mutants. The purity of GST-FOXM1 is shown on the left. **D** Schemes of FOXM1 full length and truncation mutants (top panel). In vitro binding assay by incubating the indicated Flag-FOXM1 wild type or truncation mutants bound on M2 beads with full length *Smyca* (bottom right panel). Data are mean ± SD, n = 3. *P* values are determined by one-way ANOVA with Tukey’s post hoc test. The input of FOXM1 full length and truncated mutants are shown on the bottom left panel. **E** GSEA plot for the correlation of *Smyca*-regulated transcriptome with the FOXM1 pathway signature. Enrichment score and NES are indicated. **F** The heat maps of FOXM1 ChIP-seq peaks in indicated MDA-MB-231 cell derivatives show that the peak distribution is close to the TSS. **G** Overlapping of ChIP-seq peaks from MDA-MB-231 cells stably expressing control and *Smyca* shRNAs. **H** Overlapping of *Smyca*-dependent FOXM1-bound genes with *Smyca*-upregulated genes to define *Smyca*-upregulated FOXM1 targets (left panel). GO analysis of the 96 *Smyca*-upregulated FOXM1 targets (middle panel). HR genes among the *Smyca*-upregulated FOXM1 targets and their functions in the HR pathway (right panel). The number of *Smyca*-upregulated FOXM1 targets belonging to a particular GO signature divided by the total number of genes in this signature is defined as gene ratio **I** RT-qPCR analysis of the expression of indicated FOXM1-targeted genes in control ASO- or *Smyca* ASO-transfected MDA-MB-231 cells treated with or without 10 μM olaparib for 72 h. **J** ChIRP-qPCR assay for *Smyca* occupancy on the loci of indicated FOXM1-targeted HR genes. GAPDH was used as a control. Tilling biotinylated oligonucleotides complementary to LacZ or *Smyca* were used to pull down the RNA-associated chromatins from MDA-MB-231 cells. Data are normalized with the inputs. Data in **I**, **J** are mean ± SD, n = 3. *P* values are determined by unpaired t-test

Next, we investigated the underlying mechanism by which *Smyca* stimulates HR repair. Given the strong correlation of *Smyca*-regulated transcriptome with DSB repair and HR signatures, we hypothesized that *Smyca* promotes the function of a key transcription factor that governs the transcription of a set of HR genes. To test this hypothesis, we searched for the common upstream regulators of *Smyca*-upregulated genes identified in our previous study [37] using the Upstream Regulator Analysis Tool of the Ingenuity Pathway Analysis (IPA). Using *P* < 0.05 and Z score > 1.5 as a cut-off, we identified 25 upstream regulators (Table S1, Supporting Information). Intersecting this pool of regulators with genes that are listed in the Gene ontology (GO)_DNA repair signature revealed three factors, i.e., SMARCA4, FOXM1 and TWIST1, as the potential candidates that mediate *Smyca*-promoted transcription of DNA repair genes (Fig. 3A). Among them, only FOXM1, but not SMARCA4 and TWIST1, interacted with *Smyca *in vivo, as demonstrated by RIP analysis (Fig. 3B and Figure S3A, Supporting Information). Furthermore, in vitro pull down analysis demonstrated that recombinant GST-FOXM1 interacted with in vitro transcribed *Smyca* (Fig. 3C). Using truncation mutants of *Smyca* and FOXM1, we further found that the 1–500 segment of *Smyca* and the NRD domain of FOXM1 are responsible for their interaction (Fig. 3C, D). Furthermore, GSEA analysis revealed a positive correlation between *Smyca*-regulated transcriptome and FOXM1 pathway signature (Fig. 3E). However, *Smyca* did not affect FOXM1 protein abundance (Figure S3B, Supporting Information). Thus, our study identifies FOXM1 as a *Smyca*-binding protein and suggests a potential role of *Smyca* in regulating FOXM1-governed transcription of HR genes.

To investigate the impact of *Smyca* on FOXM1-mediated transcription at a genome-wide level, we performed FOXM1 ChIP-seq analysis on MDA-MB-231 cells stably expressing control shRNA and *Smyca* shRNA and identified 12,332 peaks and 10,321 peaks, respectively. Human genomic (hg38) annotation analysis indicated that these peaks were highly enriched in the promoter regions (defined as ± 1 kb from the transcription starting site (TSS); Figure S3C, Supporting Information). Importantly, FOXM1 occupancy on the promoter regions was diminished by *Smyca* knockdown (Fig. 3F, G). Using fold change > 1.5 as a cut-off, we identified 359 genes in which FOXM1 binding was enhanced by *Smyca*. Intersecting this set of data with *Smyca*-upregulated genes from the GSE181028 dataset identified 96 FOXM1-bound genes whose expression levels were upregulated by *Smyca* (Fig. 3H, left panel and Table S2, Supporting Information). GO analysis of the 96 genes revealed multiple DNA damage/repair-related terms. Importantly, each of the three categories of “homologous recombination”, “DNA IR damage & cellular response via ATM”, and “DNA IR damage & cellular response via ATR” contains 7 HR genes, including *NBS1*, *EXO1*, *BRCA2*, *BRIP1*, *RAD51*, *POLE2*, and *RFC2* (Table S3, Supporting Information), whose actions collectively cover all steps of the HR repair pathway (Fig. 3H, middle and right panels). Conversely, the binding of FOXM1 to its NHEJ target gene *XRCC1* (Table S3, Supporting Information) was not dependent on *Smyca* (ChIP-seq fold change of shControl/sh*Smyca* = 1.1), consistent with the inability of *Smyca* to regulate NHEJ repair. RT-qPCR analysis confirmed that *Smyca* knockdown downregulated these FOXM1-bound HR genes in basal conditions. Importantly, the expression levels of most of these genes were upregulated in response to olaparib treatment and *Smyca* knockdown greatly impaired their upregulation (Fig. 3I). These findings are consistent with the induction of *Smyca* by DNA damage and demonstrated a role of *Smyca* in amplifying DDR, thereby boosting the expression of a set of HR genes.

However, *Smyca* knockdown did not affect the expression of FOXM1-dependent NHEJ gene *XRCC1* and a set of FOXM1-independent HR genes (Fig. 3I and Figure S3D, Supporting Information). To determine the dependency of FOXM1 on *Smyca*-upregulated HR genes, we generated MDA-MB-231 FOXM1 knockout (KO) cells using two independent sgRNAs (Figure S3E upper panel, Supporting Information). *Smyca* depletion in FOXM1 KO cells did not affect the transcription of FOXM1-targeted HR genes (Figure S3E lower panel, Supporting Information). Collectively, our data support an idea that *Smyca* promotes the transcription of a set of HR genes through FOXM1.

Since *Smyca* physically interacts with FOXM1, we tested whether *Smyca* enhances FOXM1 binding to the promoters of its HR target genes. ChIRP-qPCR analysis revealed the enrichment of *Smyca* in the promoters of all 7 FOXM1-targeted HR genes, but not the promoters of *Smyca*-independent FOXM1-targeted genes, including *PLK1*, *AURKB*, *CCNB1*, and *CENP* (Fig. 3J and Figure S3F and Table S3, Supporting Information). Importantly, *Smyca* recruitment to the promoters of FOXM1-targeted HR genes was still detected in FOXM1 KO cells, suggesting the independence of FOXM1 (Figure S3G, Supporting Information). Moreover, *Smyca* depletion in FOXM1 KO cells did not affect DNA DSBs in basal and cisplatin-treated conditions (Figure S3H, Supporting Information). These findings collectively indicate that *Smyca* enhances the recruitment of FOXM1 to the promoters of a set of FOXM1-targeted HR genes, thereby promoting their transcription to facilitate HR repair for maintaining genome stability.

Next, we examined the mechanism for the selectivity of *Smyca* to regulate a subset of FOXM1 targets. Using MEME motif analysis to determine the common transcription factor binding sites in the vicinity of FOXM1-binding sites on the promoters of 96 *Smyca*-dependent FOXM1 targets, we revealed the binding sites for AP-1, c-Fos, STAT3, and GCN4 as the top four enriched candidates (Figure S4A, Supporting Information). Among them, AP-1-binding site is most enriched. Importantly, inhibition of AP-1 DNA binding activity by T-5224 impaired the recruitment of both *Smyca* and FOXM1 to the promoters of 7 *Smyca*/FOXM1-dependent HR genes (Figure S4B, C, Supporting Information). However, T-5224 did not affect FOXM1 loading onto the promoters of several *Smyca*-independent FOXM1 targets that act in cell cycle regulation (Figure S4C, Supporting Information). Furthermore, inhibition of STAT3, whose binding site is also enriched in the vicinity of FOXM1-binding site on promoters of the *Smyca*-dependent FOXM1 targets (Figure S4A, Supporting Information), did not affect FOXM1 deposition to the promoters of several HR genes (Figure S4D, Supporting Information). Thus, our data suggest a sequential recruitment of AP-1, *Smyca*, and FOXM1 to the promoters of *Smyca*-dependent FOXM1 targets. However, AP-1 is dispensable for FOXM1 deposition to the promoters of *Smyca*-independent FOXM1 targets.

### *Smyca* promotes FOXM1-mediated expression of nucleotide metabolism genes for elevating dNTPs pool and maintaining genome stability

**Fig. 4:**
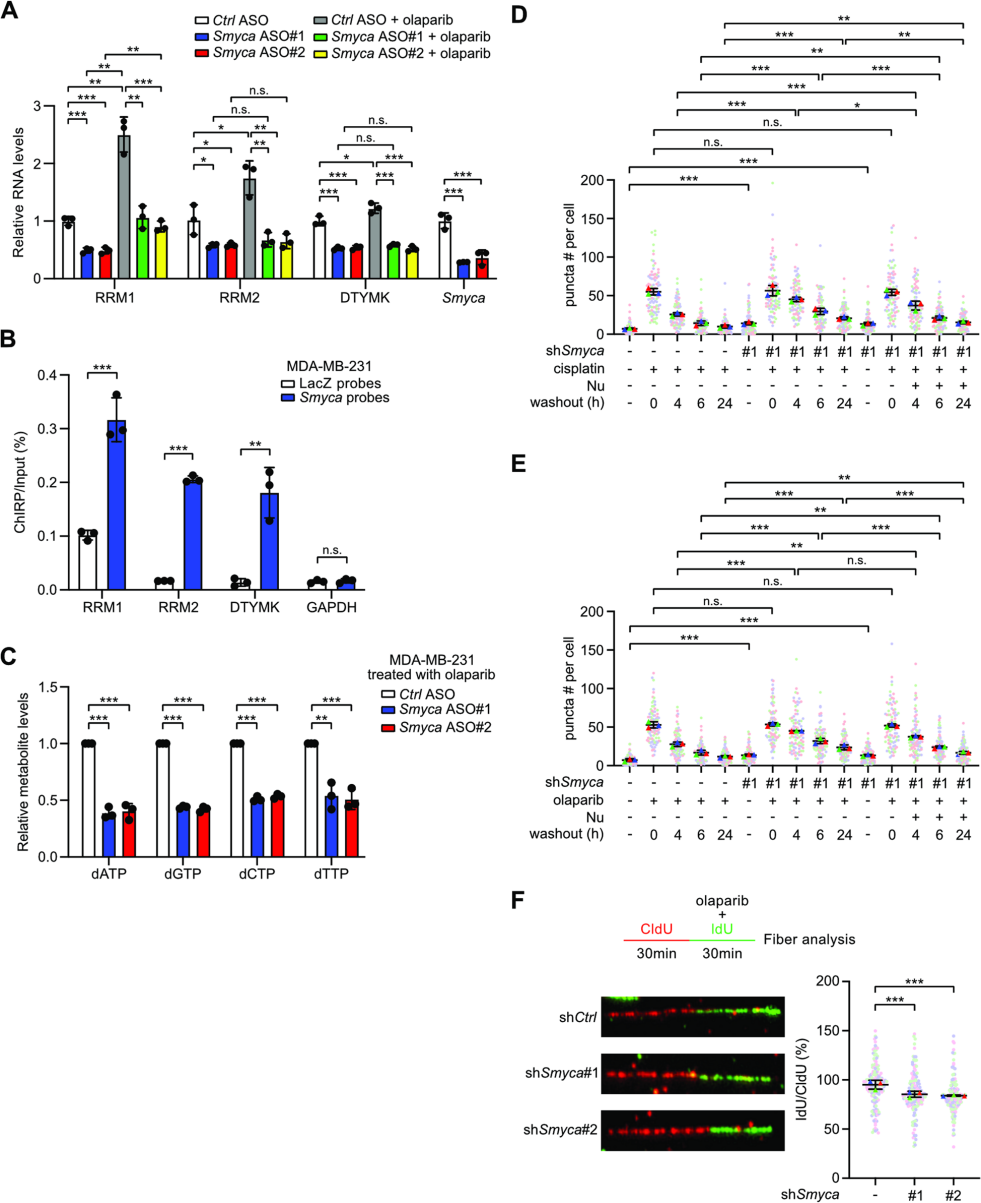
*Smyca* promotes FOXM1-mediated transcription of nucleotide metabolism genes for repairing DNA damage and preventing replication stress. **A** RT-qPCR analysis of the expression of indicated FOXM1-targeted genes in control ASO- or *Smyca* ASO-transfected MDA-MB-231 cells treated with or without 10 μM olaparib for 72 h. **B** ChIRP-qPCR assay for *Smyca* occupancy on the loci of indicated FOXM1-targeted genes. GAPDH was used as a control. **C** Rolling-circle amplification measuring dNTPs levels in indicated MDA-MB-231 cell derivatives. The knockdown efficiencies of *Smyca* ASOs are shown on the right. Data are mean ± SD, n = 3. *P* values are determined by one-way ANOVA with Tukey’s post hoc test. **D**, **E** Quantitative data for γH2AX foci in indicated MDA-MB-231 derivatives treated with 50 μM cisplatin **D** or 10 μM olaparib **E** for 4 h, followed by drug removal to allow DNA repair in the presence of absence of 12.5 μM nucleosides for indicated time periods. Data are mean ± SD, n = 3 and > 30 cells per group per experiment are analyzed. *P* values are determined by one-way ANOVA with Tukey’s post hoc test. **F** Scheme for the DNA fiber assay (top panel). Representative DNA fiber images from indicated MDA-MB-231 derivatives (bottom left panel). Quantification of the IdU/CldU ratio for indicated cells (bottom right panel). Data are mean ± SD, 50 fibers per group per experiment were analyzed. *P* values are determined by one-way ANOVA with Tukey’s post hoc test, comparing the control shRNA group with each of the two *Smyca* shRNA groups

Notably, the GO term of “pyrimidine metabolism” was also enriched in *Smyca*-dependent FOXM1 targets (Fig. 3H). This category contains several genes that participate in dNTPs synthesis, including *RRM1*, *RRM2,* and *DTYMK* (Table S2 and S3, Supporting Information). *RRM1* and *RRM2* encode the large and small subunits of ribonucleotide reductase, respectively, which catalyze a rate-limiting step in the production of DNA synthesis precursors, i.e., converting NDPs to dNDPs [49]. *DTYMK* encodes a type of thymidine kinase to convert dTMP to dTDP [50], a precursor of dTTP. RT-qPCR analysis confirmed the downregulation of *RRM1*, *RRM2*, and *DTYMK* by *Smyca* knockdown in basal and olaparib-treated conditions (Fig. 4A), whereas ChIRP-qPCR analysis showed *Smyca* recruitment to the promoters of these three genes (Fig. 4B). Furthermore, using a previously established rolling-circle amplification method to quantify cellular dNTPs pool [41], we found that *Smyca* knockdown in olaparib-treated MDA-MB-231 cells significantly decreased cellular levels of dATP, dGTP, dCTP, and dTTP (Fig. 4C). Furthermore, exogenous supplementation of nucleosides partially rescued the DSB repair defect seen in *Smyca* knockdown cells treated with cisplatin or olaparib (Fig. 4D, E). Thus, our data support a role of *Smyca* in enhancing FOXM1 recruitment to the promoters of several nucleotide metabolism genes for activating their transcription, thereby increasing the cellular dNTPs pool to contribute to the repair synthesis step of HR.

A competent HR function and a sufficient nucleotide supply are both essential for maintaining the stability and progression of replication fork [51]. Failure of this process impairs genome stability to cause resistance to chemotherapy and PARPi [52,53]. Using DNA fiber combing assay to analyze the DNA replication fork dynamics, we found that *Smyca* knockdown in olaparib-treated cells significantly decreased the fork speed (Fig. 4F). Thus, in addition to promoting the repair of DNA lesions generated by genotoxic agents, *Smyca*-mediated enhancement of HR and dNTPs pool also contribute to the maintenance of replication fork progression to avoid replication stress.

Despite the effect of *Smyca* on nucleotide pool maintenance, *Smyca* knockdown or overexpression did not affect cell proliferation (Figure S4E, F, Supporting Information), consistent with our previous study [37]. We therefore investigated the impact of *Smyca*/FOXM1 axis on cell proliferation in further details. Treatment of *Smyca* overexpressing cells with FDI-6, a small molecular inhibitor of FOXM1 [54], led to a significant decrease in cell proliferation and this effect of FDI-6 was partially rescued by exogenous supplementation of nucleosides (Figure S4F, Supporting Information). These findings support a growth-promoting effect of the *Smyca*/FOXM1 axis, which is mediated in part through the elevation of nucleotide pool. Of note, our previous study showed that *Smyca* also enhances TGF-β/Smad signaling, which elicits an anti-proliferative effect [37]. Accordingly, co-treatment of *Smyca* overexpressing cells with FOXM1 inhibitor FDI-6 and TGF-β/Smad pathway inhibitor SB431542 reverted the effect of FDI-6 on cell proliferation (Figure S4F, Supporting Information). Thus, the growth-promoting effect of *Smyca*/FOXM1 axis is neutralized by the anti-proliferative effect of *Smyca*/Smad axis, which likely explains the inability of *Smyca* to elicit a stimulatory effect on proliferation.

### *Smyca* targeting sensitizes HR-proficient TNBC cells to platinum and PARPi

**Fig. 5:**
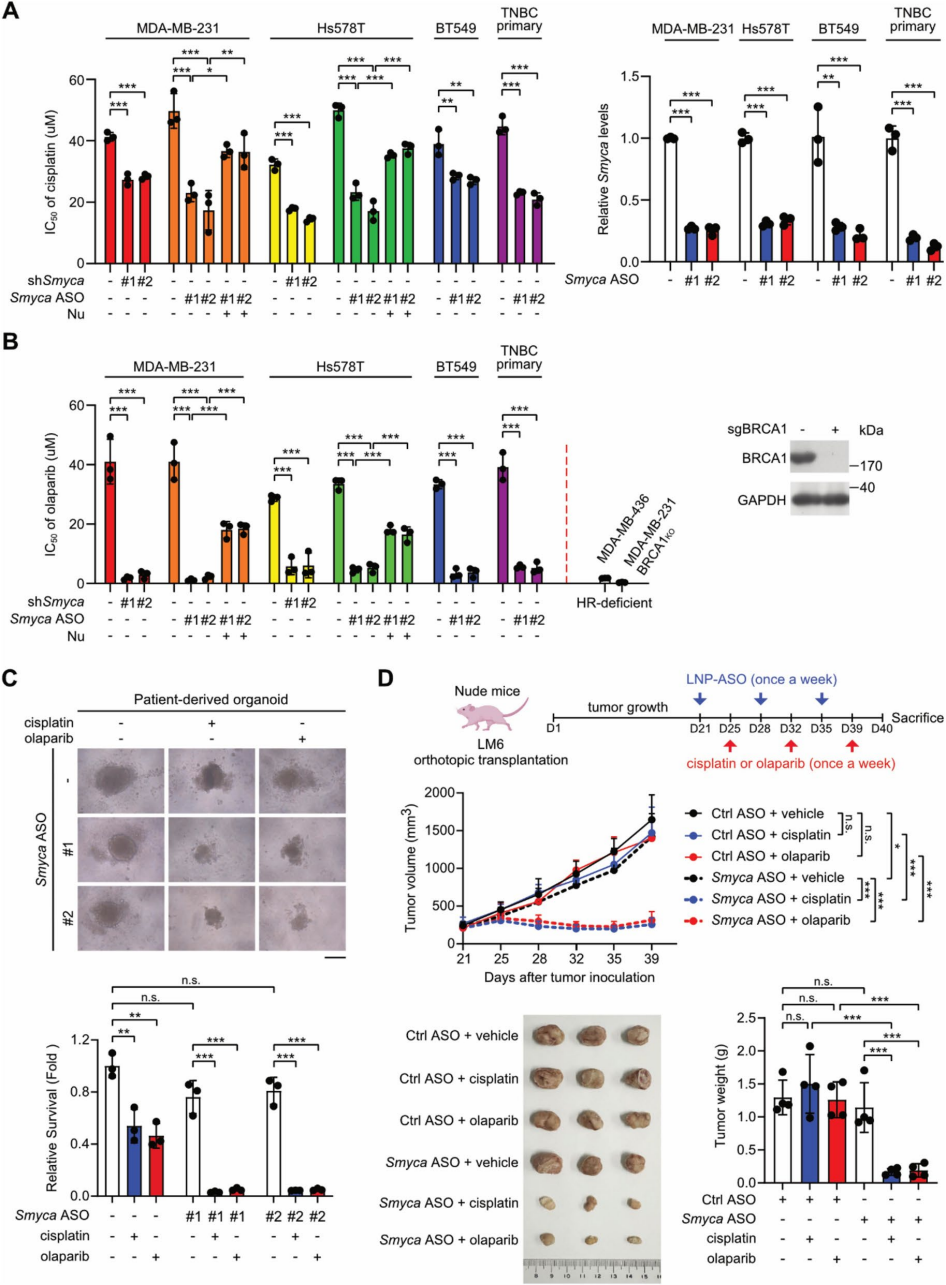
*Smyca* knockdown sensitizes TNBC cells to cisplatin and olaparib. **A** IC_50_ of cisplatin determined from indicated TNBC cell lines or TNBC primary cells stably expressing control or *Smyca* shRNAs or transfected with control or *Smyca* ASO and cultured in medium with or without 12.5 μM nucleoside supplementation. The knockdown efficiencies of *Smyca* ASOs are shown on the right. **B** IC_50_ of olaparib determined from indicated TNBC cell lines or TNBC primary cells stably expressing control or *Smyca* shRNAs or transfected with control or *Smyca* ASO cultured in medium with or without 12.5 μM nucleoside supplementation. The knockdown efficiencies of *Smyca* ASOs and the KO of BRCA1 are shown on the right or top panel, respectively. **C** Representative images (upper panel) and relative survival (lower panel) of TNBC patient-derived organoids treated with 50 μM cisplatin or 10 μM olaparib. Bar, 2 mm. **D** The scheme of nude mice orthotopically implanted with LM6 cells and treated with LNP-loaded *Smyca* ASO or control ASO, together with or without cisplatin or olaparib (top panel). Tumor volumes were measured on the indicated days and plotted on the middle panel. Tumors were surgically removed on the sacrifice day and their sizes and weights are shown on the bottom panels. Data in all panels are mean ± SD, n = 3 **A**–**C** or n = 4 **D**. *P* values were determined by one-way **A**–**C** or two-way **D** ANOVA with Tukey’s post hoc test. The characterization data of LNPs are presented in Figure S9, Supporting Information

Given a broad impact of *Smyca* on FOXM1-regulated transcription of HR and nucleotide metabolism genes, we determined whether *Smyca* targeting could increase the responsiveness of HR-proficient TNBC cells to platinum and PARPi, that is, a BRCAness phenotype. Indeed, *Smyca* knockdown by shRNAs or ASOs reduced the IC_50_ of cisplatin in TNBC cells MDA-MB-231, Hs578T, and BT549 (Fig. 5A). *Smyca* targeting by ASOs also sensitized primary TNBC cells to cisplatin (Fig. 5A). In agreement with the synthetic lethality between HR deficiency (HRD) and PARP inhibition, *Smyca* targeting in HR-proficient TNBC cell lines and primary TNBC cells dropped the IC_50_ of PARPi from 29–41 µm to 1–5 µm, approaching to the IC_50_ detected in HR-deficient TNBC cell lines (1.78 µm for MDA-MB-436 cells and 0.35 µm for MDA-MB-231 BRCA1 KO cells) (Fig. 5B). However, exogenous supplementation of *Smyca*-depleted MDA-MB-231 cells with nucleosides increased the IC_50_ of cisplatin and olaparib (Fig. 5A, B), demonstrating the contribution of dNTP pool shortage to the chemosensitization effect of *Smyca* knockdown. Furthermore, besides 2-D culture, *Smyca* targeting by ASOs also enhanced the killing effects of cisplatin and PARPi on a TNBC patient-derived organoid (Fig. 5C). Next, we tested the tumor-suppressive effect of *Smyca* targeting in combination with platinum or PARPi in vivo. Orthotopic tumors derived from MDA-MB-231 cells carrying control and *Smyca* shRNA were grown at a similar rate. However, while the former tumors were virtually resistant to cisplatin or olaparib therapy, the growth of tumors derived from *Smyca*-knockdown MDA-MB-231 cells were almost completely suppressed by cisplatin or olaparib treatment (Figure S5A, Supporting Information). To develop a clinically more applicable approach of *Smyca* targeting, we employed *Smyca*-specific gapmer ASO loaded onto lipid nanoparticles (LNPs), which was shown to successfully knockdown *Smyca* [37]. In addition, we utilized the orthotopic model derived from LM6 cells, a highly aggressive variant of MDA-MB-231 [55]. After tumor growth, ASO-loaded LNPs and cisplatin or olaparib were injected alternately (Fig. 5D). Again, *Smyca* ASO greatly sensitized LM6-derived tumors to cisplatin and olaparib. IHC analysis revealed that tumors derived from combined treatment displayed reduced proliferating nuclei (Ki67) staining but increased γH2AX and active caspase 3 stainings, compared with those derived from cisplatin or olaparib single treatment (Figure S5B, Supporting Information). Furthermore, while tumors derived from cisplatin- or olaparib-treated mice expressed a higher level of several FOXM1-targeted HR genes, this was greatly suppressed in those derived from combined treatment (Figure S5C, Supporting Information). Thus, our data indicate that *Smyca* targeting sensitizes HR-proficient TNBC tumors to platinum and PARPi, thus creating a BRCAness phenotype.

### *Smyca* targeting in combination with platinum or PARPi activates cGAS-STING pathway and tumor immunogenicity

**Fig. 6:**
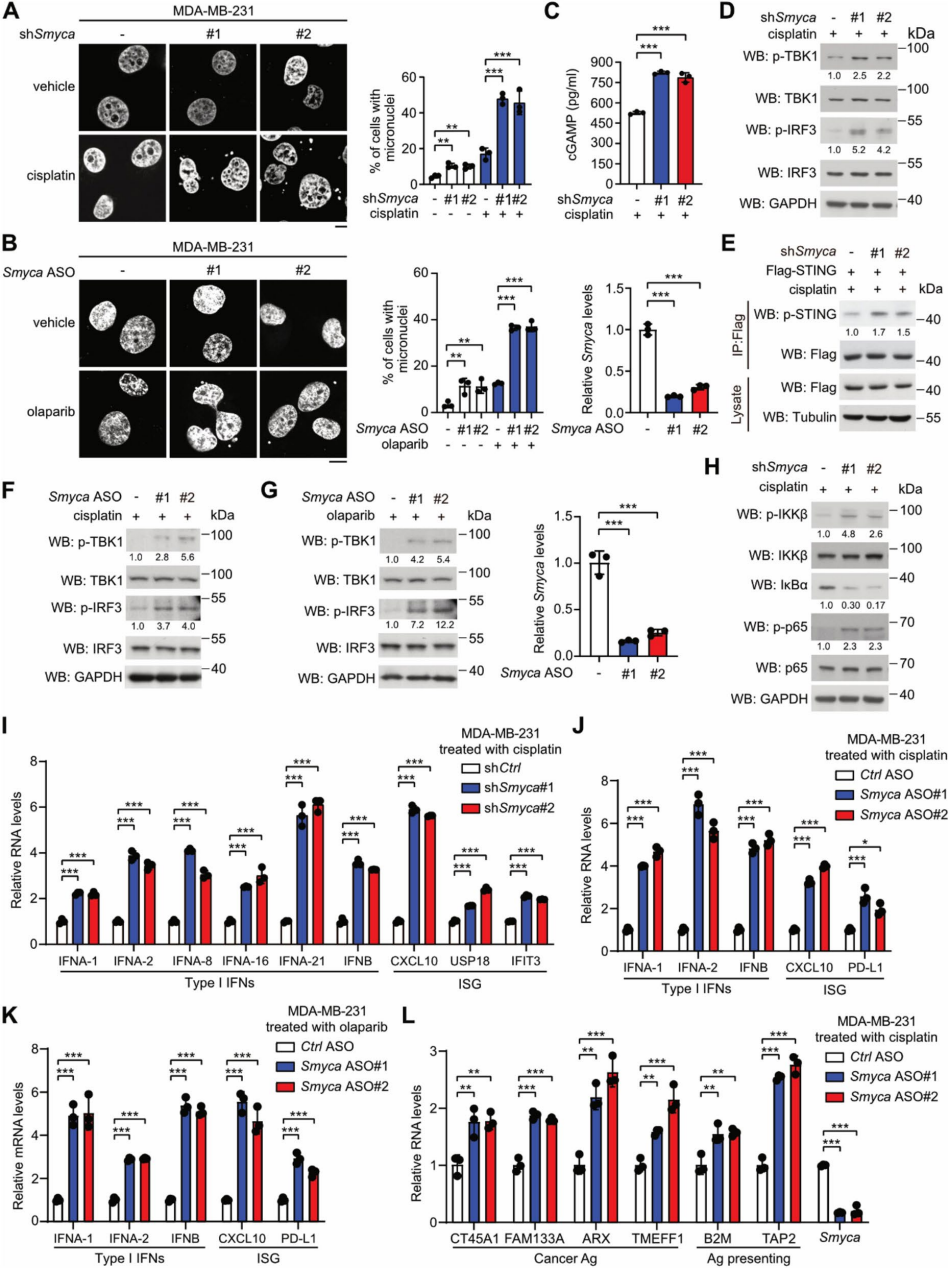
*Smyca* targeting in combination with cisplatin or olaparib treatment activates cGAS-STING pathway and tumor immunogenicity. **A**, **B** Micronuclei were stained by DAPI in MDA-MB-231 cells stably expressing control or *Smyca* shRNAs and treated with 50 μM of cisplatin **A** or 10 μM of olaparib **B** for 48 h. Bar, 10 μm. Data are mean ± SD, n = 3 and > 30 cells per group per experiment are analyzed. *P* values are determined by one-way ANOVA with Tukey’s post hoc test. The knockdown efficiencies of *Smyca* ASOs are shown on the right panel of **B**. **C** cGAMP level in MDA-MB-231 cells stably expressing control or *Smyca* shRNAs and treated with 50 μM of cisplatin for 72 h. **D**, **F**–**H** Western blot analysis of indicated proteins in MDA-MB-231 cells stably expressing control or *Smyca* shRNAs and treated with 50 μM cisplatin for 72 h **D**, **H**, or MDA-MB-231 cells transfected with control or *Smyca* ASOs and treated with 50 μM cisplatin **F** or 10 μM olaparib **G** for 72 h. The knockdown efficiencies of *Smyca* ASOs are shown on the right panel of **G**. **E** Flag-STING was immunoprecipitated from MDA-MB-231 cells stably expressing control or *Smyca* shRNAs, treated with 50 μM of cisplatin for 72 h and transfected with Flag-STING, followed by Western blot analysis with indicated antibodies. **I**–**L** RT-qPCR analysis of indicated genes in MDA-MB-231 cells stably expressing control or *Smyca* shRNAs treated with 50 μM of cisplatin for 72 h **I**, or MDA-MB-231 cells transfected with control or *Smyca* ASOs and treated with 50 μM cisplatin **J**, **L**, or 10 μM of olaparib **K** for 72 h. Data in **C**, **G**, **I**, **J**, **K**, **L** are mean ± SD, n = 3. *P* values are determined by one-way ANOVA with Tukey’s post hoc test. The quantifications in (D-G) are the intensity ratios of phospho/total protein normalized to loading control

Given a critical role of HRD in the induction of anti-tumor immune surveillance, we examined the possibility of *Smyca* targeting for the induction of such responses. *Smyca* knockdown by shRNAs or ASOs in MDA-MB-231 cells enhanced micronuclei formation under basal and cisplatin- or olaparib-treated conditions (Fig. 6A, B and Figure S6A, Supporting Information). Similar effects were observed by *Smyca* knockdown in Hs578T cells (Figure S6B, Supporting Information). Given the synergistic effect of combined *Smyca* targeting with platinum or PARPi on the killing of HR-proficient TNBC, we focused on the combined therapy. Importantly, *Smyca* knockdown by shRNAs or ASOs in combination with cisplatin or olaparib treatment increased the cellular cGAMP levels as well as the levels of p-TBK1, p-IRF3, and p-STING in MDA-MB-231 cells (Fig. 6C–G), demonstrating the activation of cGASSTING axis. Similar effects were observed in Hs578T cells (Figure S6C-E, Supporting Information). However, *Smyca* knockdown in combination with cisplatin did not affect the mRNA levels of cGAS and STING (Figure S6F, Supporting Information). In addition, combined *Smyca* targeting with cisplatin treatment in MDA-MB-231 or Hs578T cells increased IKKβ and p65 phosphorylation, IκB degradation, and p65 nuclear translocation (Fig. 6H and Figure S6G-I, Supporting Information), consistent with the activation of NF-κB pathway by cGASSTING signaling. Consequently, combined *Smyca* targeting with cisplatin or olaparib treatment induced the expression of type I IFN genes and IFN-stimulatory genes (ISGs), and enhanced the secreted IFNβ and the IFNβ reporter activity (Fig. 6I–K and Figure S6J-L, Supporting Information), thus demonstrating the activation of type I IFN responses. Importantly, the induction of IFNβ and ISG by *Smyca* targeting in combination with cisplatin was abrogated by cGAS inhibitor, G140 (Figure S6M, Supporting Information), supporting that *Smyca* knockdown-stimulated type I IFN responses requires the activation of cGAS-STING axis. *Smyca* targeting in combination with cisplatin or olaparib treatment also induced PD-L1 (Fig. 6J, K), which is a target of both NF-κB and IFN [56,57]. Although induction of PD-L1 in tumor cells causes T cells exhaustion and immune evasion, high PD-L1 expression also correlates with increased sensitivity to immune checkpoint blockade (ICBs) [58]. Together, our study demonstrated that *Smyca* targeting in combination with platinum or PARPi activates cGASSTING pathway to stimulate type I IFN responses.

FOXM1 functional impairment and PARPi are both capable of inducing the expression of a set of cancer antigens and antigen processing/presenting genes [18,59]. Accordingly, *Smyca* targeting in combination with cisplatin or olaparib treatment of MDA-MB-231 cells enhanced the expression of cancer antigen genes *CT45A1*, *FAM133A*, *ARX*, *TMEFF1*, and antigen presenting genes *B2M* and *TAP2* (Fig. 6L and Figure S6N and Table S3, Supporting Information). These data suggest that combined *Smyca* targeting with platinum or PARPi stimulates tumor immunogenicity.

### FOXM1 targeting in combination with platinum or PARPi activates cGAS-STING pathway

Since FOXM1 is a key mediator of *Smyca*-promoted HR repair, FOXM1 targeting should resemble the effects of *Smyca* targeting. Consistent with this notion, FOXM1 ablation sensitizes HR-proficient TNBC cells to PARPi [17]. We further investigated whether FOXM1 targeting in combination with cisplatin or olaparib could activate cGASSTING pathway. Importantly, FOXM1 KO induced micronuclei formation and this effect was further aggravated by treating the KO cells with cisplatin or olaparib (Figure S7A-B, Supporting Information). Furthermore, FOXM1 KO in combination with cisplatin or olaparib boosted the levels of p-TBK1 and p-IRF3 and the expression of type I IFNs and ISG (Figure S7C-F, Supporting Information), indicating the activation of cGASSTING axis and type I IFN responses. The FOXM1 inhibitor FDI-6 was previously tested in human TNBC cells [54]. We found that FDI-6 treatment of mouse TNBC cell line 4T1 reduced the expression of Foxm1-targeted HR genes, such as *Nbs1*, *Brip*, *Brca2*, and *Rad51*, and the activity of Foxm1 reporter (Figure S7G-H, Supporting Information), confirming the ability of FDI-6 to inhibit Foxm1 in mouse. As a result, treatment of 4T1 cells with FDI-6 together with cisplatin or olaparib increased the phosphorylation of Tbk1 and Irf3 (Figure S7I-J, Supporting Information). Our data support that FOXM1 targeting in combination with platinum or PARPi activates cGAS-STING pathway.

### *Smyca* or FOXM1 targeting in combination with platinum or PARPi promotes anti-tumor immunity

**Fig. 7:**
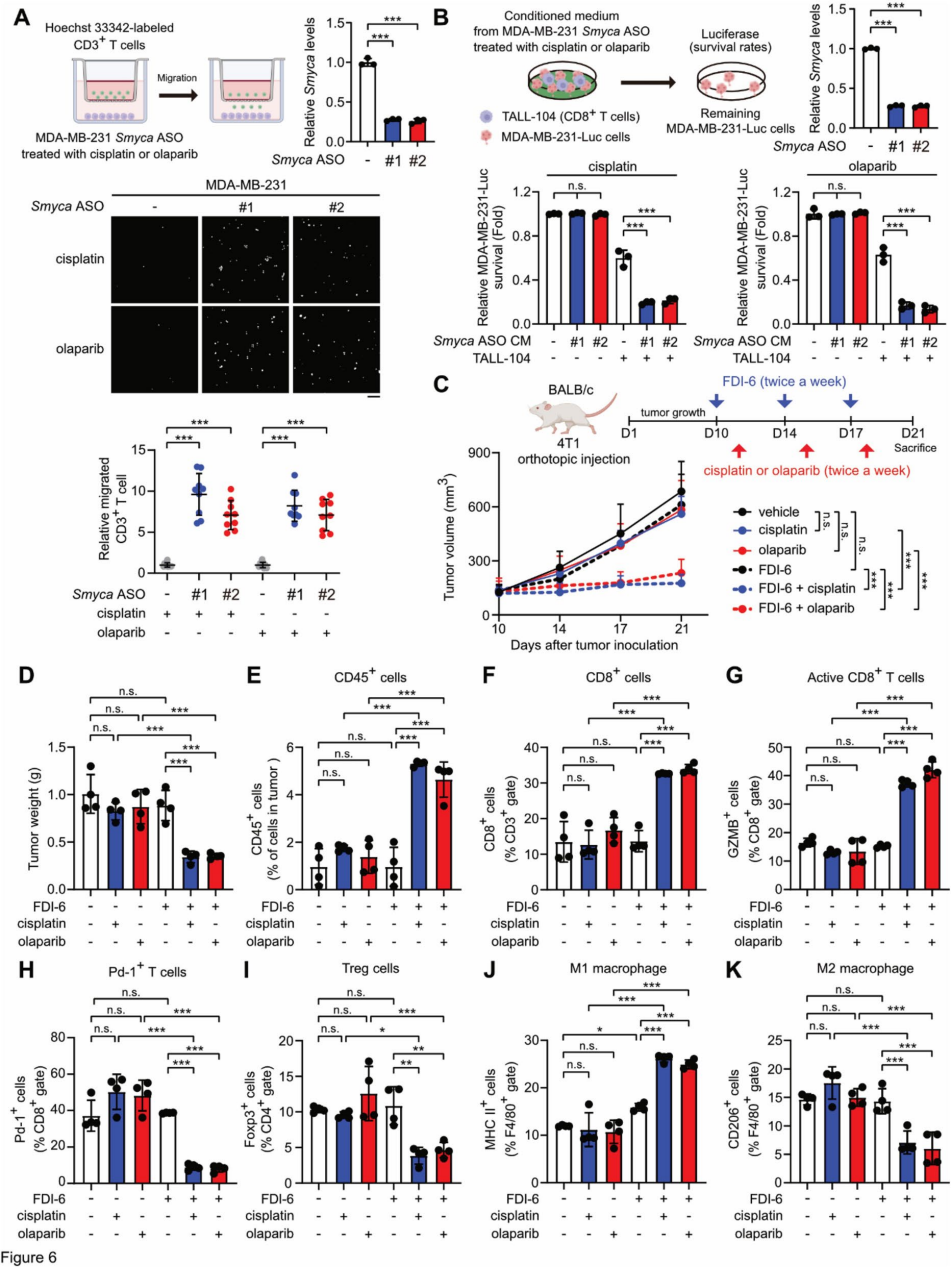
*Smyca* or FOXM1 targeting in combination with cisplatin or olaparib treatment promotes anti-tumor immunity. **A** Upper left panel: Scheme of the transwell T cell migration assay. Upper right panel: The knockdown efficiencies of *Smyca* ASOs. Middle and bottom panels: Representative images of Hoechst 33342-labeled CD3^+^ T cells (middle) and relative amounts of migrated CD3^+^ T cells in the lower chambers (bottom). Bar, 200 μm. Data are mean ± SD, n = 9. *P* values are determined by one-way ANOVA with Tukey’s post hoc test. **B** Upper left panel: Scheme of the T cell-mediated tumor cell killing assay. Upper right panel: The knockdown efficiencies of *Smyca* ASOs. Bottom panels: Relative amounts of luminescence derived from survived MDA-MB 231-Luc cells after co-cultured with CD8^+^ T cells in the presence of indicated CM. Data are mean ± SD, n = 3. *P* values are determined by one-way ANOVA with Tukey’s post hoc test. **C** Top panel: The scheme of BALB/C mice orthotopically implanted with 4T1 cells and treated with FDI-6, cisplatin, olaparib, and/or vehicle. Bottom panel: Tumor volumes were measured on the indicated days. **D** Tumor weights of experiment shown in **C** determined on the sacrifice day. **E**–**K** Flow cytometry analysis for the percentages of indicated types of TILs in the tumor tissues derived from **C**. Data in **C**–**K** are mean ± SD, n = 4. *P* values are determined by two-way ANOVA with Tukey’s post hoc test

Given the activation of cGASSTING pathway and tumor immunogenicity by targeting the *Smyca*/FOXM1 axis in combination with cisplatin or olaparib, we thought to determine the effect of combined treatment on tumor immune microenvironment. First, IHC staining of the TNBC tumors derived from mice treated with LNP-loaded *Smyca* ASO together with cisplatin or olaparib contained a higher amount of infiltrating M1-like macrophages but a lower amount of M2-like macrophages, compared with single agent or control agent-treated mice (Figure S5B, Supporting Information). This finding is consistent with a role of cGASSTING pathway in promoting macrophage M1 polarization [60]. However, the absence of *Smyca* in mouse [61] precludes us to assess the effect of combined treatment on tumor-infiltrating T cells in a syngeneic mouse model. We therefore turned to the ex vivo system. First, we monitored the effect of T cell recruitment to tumor cells using Transwell migration assay. Notably, combined treatment of MDA-MB-231 cells with *Smyca* ASOs and cisplatin or olaparib induced the migration of a higher amount of CD3^+^ T cells towards the tumor cells, as compared with single agent- or control agent-treated group (Fig. 7A). To assess the T cell-mediated tumor killing effect, we used the ex vivo co-culture of MDA-MB-231-Luc cells (MDA-MB-231 cells stably expressing firefly luciferase) with human leukemic T cell line TALL-104, which expresses CD8^+^ T and NK markers and kills tumor cells through a non-MHC restricted manner [62]. Conditioned medium (CM) derived from MDA-MB-231 cells co-treated with *Smyca* ASOs and cisplatin or olaparib elicited a stronger effect to stimulate TALL-104-mediated tumor cell killing, as demonstrated by the reduced luciferase activities (Fig. 7B). Together, our study demonstrated the induction of anti-tumor immune surveillance by combined *Smyca* targeting with cisplatin or olaparib treatment.

Due to the lack of *Smyca* in mouse, we chose to target Foxm1 by FDI-6 in 4T1-derived tumors from a syngeneic mouse model as a surrogate approach to project the effect of targeting *Smyca*/FOXM1 axis on tumor immune microenvironment in vivo. FDI-6 in combined with cisplatin or olaparib treatment greatly suppressed tumor growth, while cisplatin or olaparib alone showed little effect (Fig. 7C, D and Figure S7K, Supporting Information). Next, we analyzed TILs using multicolor flow cytometry (see Figure S8, Supporting Information for gating strategies). Compared with untreated or single agent-treated tumors, combined treatment increased the tumor-infiltrating total leukocytes (CD45 + cells), CD8 + T cells, active CD8 + T cells (granzyme B + /CD8 +), but decreased exhausted CD8 + T cells (Pd-1 + /CD8 +) and Treg cells (Fig. 7E–I). Combined treatment also increased the tumor-infiltrating M1-like macrophages but decreased M2-like macrophages (Fig. 7J, K). Together, our findings support that FOXM1 targeting in combined with platinum or PARPi treatment reshapes an anti-tumor immune microenvironment.

### Clinical association of *Smyca*/FOXM1 axis with HR repair, therapy resistance and immune suppressive tumor microenvironment

**Fig. 8:**
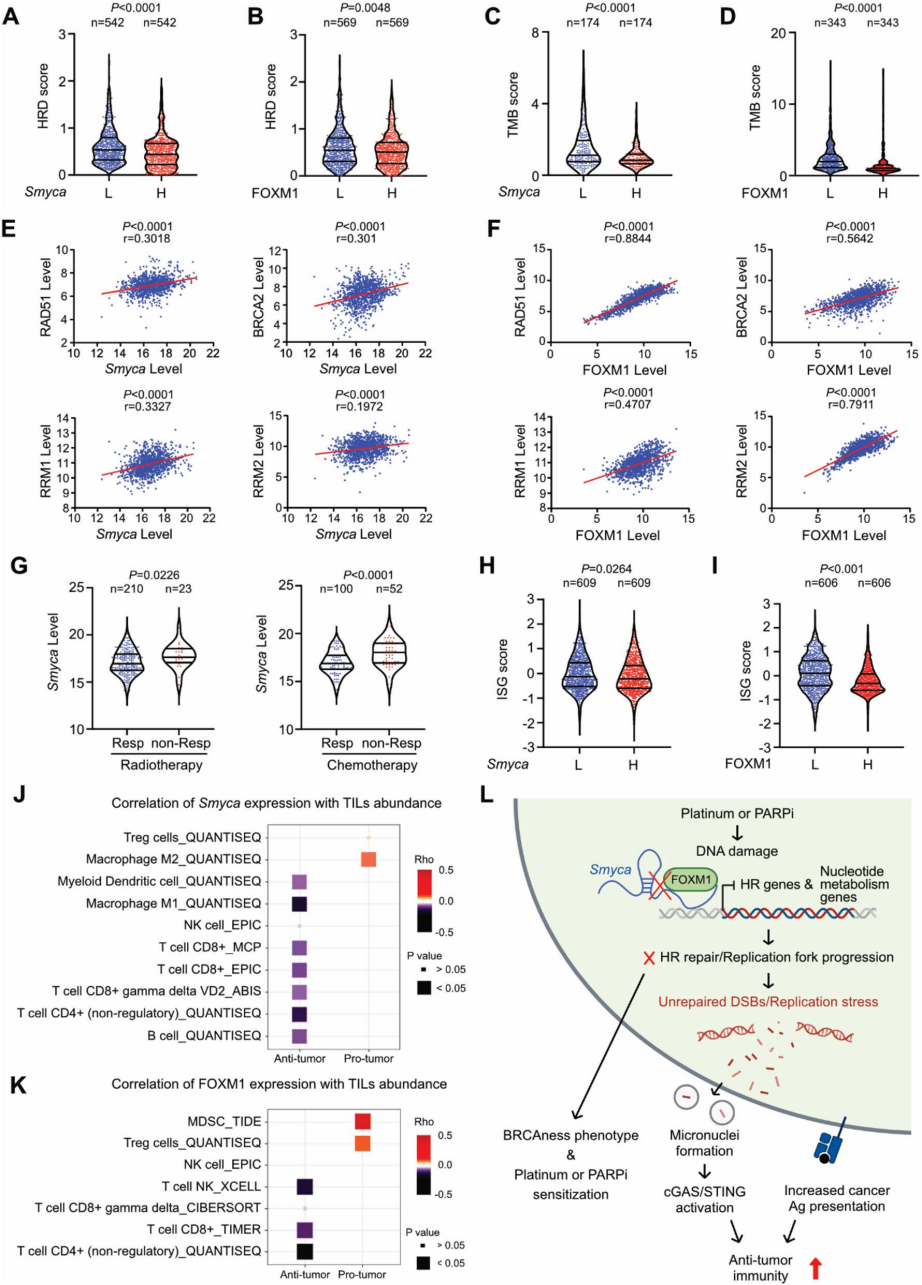
*Smyca* and *FOXM1* expression in breast cancer patients correlates positively with HR and therapy resistance and negatively with anti-tumor immunity. **A**, **B** The HRD score in the TCGA BRCA cohort according to their *Smyca* **A** or *FOXM1* **B** expression status. **C**, **D** The TMB score in the TCGA BRCA cohort according to their *Smyca* **C** or *FOXM1* **D** expression status. **E**, **F** Representative correlation plots of *Smyca* **E** or *FOXM1* **F** expression with the expression of indicated genes by analyzing BRCA datasets from TCGA (n = 1099). Pearson’s coefficients (r) and *P* values are shown. **G** The responsiveness toward radiotherapy (Left panel) or chemotherapy (Right panel) in the TCGA BRCA cohort in relation to their *Smyca* expression status. **H**, **I** The ISG signature score in the TCGA BRCA cohort according to their *Smyca* **H** or *FOXM1* **I** expression status. **J**, **K** The heat map showing the correlation of *Smyca* **J** or *FOXM1* **K** expression levels with the infiltration of indicated anti-tumor or pro-tumor immune cells. Correlations were calculated through the *Immunedeconv* R package. *P* value are determined by Spearman’s correlation. **L** Schematic presentation of the targeting of *Smyca*-FOXM1 axis to block HR repair and replication fork progression for inducing BRCAness and to activate cGAS/STING pathway and tumor antigen presentation for inducing anti-tumor immunity

Having demonstrated the role of *Smyca*/FOXM1 axis in HR repair to induce chemoresistance and immune evasion, we next assessed the clinical relevance of these findings. First, we retrieved genomic data from TCGA breast cancer database to calculate the HRD score and Tumor mutation burden (TMB) score. Patients with a high expression of *Smyca* or *FOXM1* displayed lower HRD and TMB scores (Fig. 8A–D). In addition, analysis of the transcriptomic data from breast cancer patients revealed that the expression levels of *Smyca* or *FOXM1* correlated with that of *RAD51*, *BRCA2*, *RRM1*, and *RRM2*, key targets of FOXM1 that contribute to HR and nucleotide metabolism (Fig. 8E, F). In line with these findings, patients who failed to respond to radiotherapy or chemotherapy expressed higher levels of *Smyca*, compared with the responders (Fig. 8G). Furthermore, using the expression profile of 38 ISG genes to define ISG score [63], we found that high expression of either *Smyca* or *FOXM1* correlated with a lower ISG score (Fig. 8H, I). To estimate the immune cell profile in tumor microenvironment, we utilized *Immunodeconv* R package [64] to analysis the tumor transcriptomes from TCGA breast cancer database and found that *Smyca* and *FOXM1* expression correlated negatively with the infiltration of anti-tumor immune cells but positively with the infiltration of pro-tumor immune cells (Fig. 8J, K). These findings identify a positive correlation of *Smyca*/FOXM1 axis with HR repair and therapy resistance and a negative correlation with type I IFN responses and anti-tumor immune microenvironment in breast cancer patients.

## Discussion

Our previous study identified *Smyca* as an oncogenic lncRNA by eliciting multiple tumor-promoting functions, including EMT and EMT-associated metastasis, stemness, and chemoresistance [37]. In this study, we uncover additional mechanisms that contribute to the chemoresistance function of *Smyca*, i.e., promoting HR repair and preventing replication stress (Fig. 8L). These effects are mediated in part through the ability of *Smyca* for promoting FOXM1 recruitment to the promoters of 7 HR genes and 3 nucleotide metabolism genes, thereby inducing their transcription. Importantly, the functions of these 7 HR gene products cover all steps in the HR repair pathway, including the key HR proteins RAD51 and BRCA2, whereas the three nucleotide metabolism gene products are important for maintaining cellular dNTPs pool needed for repair synthesis. Besides the important impact on DNA repair, HR activity and dNTP pool are crucial for maintaining the stability and progression of replication fork during replication [51,65]. Accordingly, *Smyca* ablation in olaparib-treated TNBC cells decreases replication fork progression rate. Given the dual roles in HR repair and replication fork progression, our study identifies *Smyca* as a key player in maintaining genome stability to cause chemoresistance.

Importantly, the aforementioned therapy resistance functions of *Smyca* are not only demonstrated by cell and animal studies, but also supported by clinical data. *Smyca* expression in breast cancer patients correlates positively with HR repair and therapy resistance. Thus, the preclinical and clinical evidence derived from our study highlights a great potential of *Smyca* targeting in TNBC therapy. Notably, RNA therapeutics have been an emerging class of strategy for disease intervention, including cancer [66], and lncRNAs are among the promising targets of cancer therapy [35]. We show that *Smyca* ASO treatment of HR-proficient TNBC cells decreases the IC_50_ of olaparib to the level resembling that of HR-deficient TNBC cells, indicating the induction of a BRCAness phenotype. The ability of *Smyca* ASO to promote BRCAness implies its boarder therapeutic utilities in TNBC and ovarian cancers, including the sensitization of HR-proficient tumors to PARPi, platinum derivatives, and topoisomerase 1 inhibitors, the prevention of HR-deficient tumors to develop PARPi resistance, and the re-sensitization of certain resistant tumors to PARPi [6,8,67,68].

Besides chemosensitization, *Smyca* ablation in platinum- or PARPi-treated TNBC cells also activates cGAS/STING pathway and enhances tumor immunogenicity (Fig. 8L). These functions of *Smyca* are in line with recent reports for the profound roles of FOXM1 in shaping an immunosuppressive tumor microenvironment [18,69]. Clinically, *Smyca* expression correlates negatively with high TMB, IFN signature, and an “immune hot” tumor microenvironment. All these features are associated with an improved response to ICBs [70]. Furthermore, *Smyca* targeting in combination with platinum or PARPi induces PD-L1 expression. Although PD-L1 upregulation in tumor cells promotes T cell exhaustion and immune evasion, it also sensitizes tumors to ICB therapy [58]. Thus, our findings implicate a potential of combined *Smyca* ASO with platinum or PARPi in sensitizing HR-proficient TNBC and ovarian cancers to immunotherapy.

Notably, a previous study revealed the upregulation of PD-L1 by FOXM1 [71]. However, perhaps due to a very low basal expression level, PD-L1 is not detected by our transcriptomics analysis of control or *Smyca* knockdown cells (GSE181028). Thus, it remains unclear whether PD-L1 is a *Smyca*-dependent FOXM1 target. On the contrary to a potential positive role of *Smyca* in regulating FOXM1-mediated PD-L1 expression, we show that *Smyca* depletion under DNA damage activates cGAS/STING pathway to enhance PD-L1 expression. Thus, even if *Smyca* depletion could diminish the basal level expression of PD-L1, this effect is greatly surpassed by the robust cGAS/STING activation and PD-L1 induction under DNA damage condition.

We show that *Smyca* expression is induced by genotoxic agents through an ATM/ATR/DNA-PK-dependent mechanism, and AP-1 likely acts downstream of these DNA damage-sensing kinases to induce *Smyca* expression. These findings, together with the induction of HR gene expression by the *Smyca*/FOXM1 axis, suggests that *Smyca* participates in a feedback mechanism to amplify DDR for boosting the expression of several HR players. In support of this notion, we show that DNA damage elevates the expression of several FOXM1-trageted HR genes, consistent with previous reports [72,73]. Furthermore, *Smyca* ablation greatly compromises this DNA damage-induced HR genes expression, thus highlighting the importance of *Smyca* in promoting genotoxic stress-induced HR repair and the maintenance of genome stability. It would be intriguing to determine whether *Smyca* expression is induced in patients with acquired resistance to PARPi and other chemotherapeutic agents and whether such induction contributes to the acquired resistance.

Interestingly, *Smyca* affects FOXM1 recruitment to only a subset set of its target promoters. FOXM1 binding to the promoters of several G2/M regulators, such as *PLK1*, *AURKB*, *CCNB1*, *CENP*, and the NHEJ player *XRCC1* is not dependent on *Smyca.* The latter enables the effect of *Smyca* on DSB repair to be restricted to the error-free HR repair without affecting the error-prone NHEJ repair, thereby enhancing genome stability. The *Smyca*-dependent FOXM1 targets also do not include many known FOXM1 targets acting in invasion, metastasis, and angiogenesis [18–20,74–77], as no GO terms related to these functions were recovered (Fig. 3H). The preference of *Smyca’s* action towards HR repair-related FOXM1 targets implies that *Smyca* targeting might lead to less unwanted side effects than FOXM1 targeting. Furthermore, although several small molecular inhibitors of FOXM1 have been developed, none of them have reached to the clinical trial [78]. *Smyca* targeting by ASO could offer an alternative strategy.

The specificity of *Smyca* to influence only a subset of FOXM1 targets may be resulted from a selective recruitment of *Smyca* to the promoter regions of these genes. It would require ChIRP-seq analysis of *Smyca* genome-wide binding landscape to substantiate this idea. In addition, even if this is the case, it remains unclear how *Smyca* is recruited to these promoters. One possibility is that *Smyca* is recruited indirectly via certain chromatin-remodeling factors, transcription coactivator, or transcription factors. To explore the last possibility, we used MEME motif analysis to determine the common transcription factor-binding sites in the vicinity of FOXM1-binding sites on promoters of the 96 *Smyca*-regulated FOXM1 targets, and revealed AP-1 as the top one candidate. Importantly, inhibition of AP-1 DNA binding impairs the recruitment of both *Smyca* and FOXM1 to the promoters of 7 *Smyca*-dependent FOXM1 targets that act in HR, without affecting FOXM1 deposition to the promoters of *Smyca*-independent FOXM1 targets that act in G2/M control. These findings not only suggest a role of AP-1 in determining the selectivity of *Smyca* towards a subset of FOXM1 targets, but support a sequential loading of AP-1, *Smyca*, and FOXM1 onto the HR gene promoters. Of note, the recruitment of FOXM1 to the promoters of G2/M genes requires the MuvB/B-Myb complex, which functions as a pioneer factor to remodel nucleosomes, thereby exposing nucleosomal DNA for FOXM1 binding [79]. AP-1 is also a pioneer factor, which recruits chromatin remodeling factor, such as the SWI/SNF complex, to turn an inactive chromatin state into an open and accessible chromatin [80]. Further studies are required to determine whether AP-1 functions as a pioneer factor to facilitate the subsequent loading of *Smyca* and FOXM1.

FOXM1 is known to play a crucial role in cell cycle progression [21]. However, this and our previous [37] studies show that *Smyca* does not elicit a stimulatory effect on cell proliferation. One reason for this discrepancy is that *Smyca* fails to regulate many FOXM1 targets that control cell cycle progression. Nevertheless, *Smyca* promotes the transcription of FOXM1 targets that act on nucleotide metabolism to elevate the cellular dNTP pool. We show that *Smyca*/FOXM1 axis elicits a promotion effect on cell proliferation, which is mediated in part through the dNTP pool elevation. Nevertheless, the positive effect of *Smyca*/FOXM1 axis on cell proliferation is neutralized by the strong anti-proliferative effect of *Smyca*/Smad axis, and thus the net effect of *Smyca* on proliferation is not significantly observed.

## Conclusions

Our study identifies a key role of lncRNA *Smyca* in enhancing FOXM1-mediated transcription of a set of HR and nucleotide metabolism genes, thereby promoting HR repair. *Smyca* targeting induces BRCAness and anti-tumor immune surveillance and is therefore a potential strategy to sensitize repair-proficient TNBC to chemotherapy, PARPi, and immunotherapy.

## Supplementary Information

Supplementary Material 1

## Data Availability

The ChIP-seq data are deposited to the Gene Expression Omnibus (GEO) database with the project accession number GSE298060. Any additional information required to reanalyze the data reported in this paper is available from the corresponding authors upon request.

## Abbreviations

TNBC: Triple-negative breast cancer
HR: Homologous recombination
ChIP-seq: Chromatin immunoprecipitation sequencing
ChIRP: Chromatin isolation by RNA purification
dNTP: Deoxyribonucleoside triphosphates
DDR: DNA damage response
PARPi: PARP inhibitor
NHEJ: Non-homologous end joining
LLPS: Liquid–liquid phase separation
DSB: DNA double strand breaks
IFN: Interferon
LncRNA: Long non-coding RNA
*Smyca*: Smad/Myc coactivator
IPA: Ingenuity pathway analysis
GO: Gene ontology
RIP: RNA immunoprecipitation
TSS: Transcription starting site
KO: Knockout
LNP: Lipid nanoparticle
IHC: Immunohistochemistry
ISG: IFN-stimulatory gene
CM: Conditioned medium
HRD: HR deficiency
TMB: Tumor mutation burden
PBMC: Peripheral blood mononuclear cell
IL-2: Interleukin-2
DAB: Diaminobenzidine tetrahydroxychloride
BRCA: Breast cancer
ICB: Immune checkpoint blockade
