# Supplementary material for: Smyca-FOXM1 ribonucleoprotein complex promotes homologous recombination and tumor immune evasion to define a therapeutic target of triple-negative breast cancer
Source: J Biomed Sci. 2026 Aug 11;33:81. doi: 10.1186/s12929-026-01279-2 (PMC13459298; doi:10.1186/s12929-026-01279-2)
Supplement: Supplementary file 1 [file 12929_2026_1279_MOESM1_ESM.docx]

Supplementary Materials for

***Smyca*-FOXM1 ribonucleoprotein complex promotes homologous recombination and tumor immune evasion to define a therapeutic target of triple-negative breast cancer**

Han-Hsiun Chen *et al.*

*Correspondence: [hyichen@tmu.edu.tw](mailto:hyichen@tmu.edu.tw); [rhchen@gate.sinica.edu.tw](mailto:rhchen@gate.sinica.edu.tw)

**This PDF file includes:**

Figs. S1 to S9

Tables S1 to S6

Reference 1

**Fig. S1. *Smyca* deficiency impairs HR repair.** (A) GSEA with Hallmark Pathway analysis of DEGs based on RNA‑seq data derived from MDA‑MB‑231 cells stably expressing control shRNA and *Smyca* shRNA. (B, D) RT-qPCR analysis of *Smyca* expression levels in Hs578T cells treated with 50 μM cisplatin or 10 μM olaparib for 72 h or in MDA-MB231 cells treated with 50 μM cisplatin or 10 μM olaparib for 72 h together with or without 20 μM AP-1 inhibitor T-5224 for 24 h (D). Data are mean ± SD, n=3. *P* values are determined by one-way ANOVA with Tukey’s post hoc test. (C) Top panel: The gene annotation of *Smyca* gene locus with AP-1 binding sites marked. Bottom panel: The AP-1 ChIP-seq data from MCF-7 cells in the ReMap ChIP-seq tracks on the UCSC Genome Browser. (E, F) Representative confocal images of RAD51 (E) and BRCA1 (F) foci in indicated Hs578T derivatives treated with 50 μM cisplatin for 4 h (for BRCA1 foci) or 2 h (for RAD51 foci). Bar, 10 μm. Data are mean ± SD, n=3 and >30 cells per group per experiment are analyzed. *P* values are determined by one-way ANOVA with Tukey’s post hoc test.

**Fig. S2. *Smyca* deficiency impairs DSB repair.** (A) Representative confocal images and quantitative data for γH2AX foci in indicated Hs578T cell derivatives treated with 50 μM cisplatin for 4 h, followed by drug removal to allow DNA repair for indicated time periods. Bar, 10 μm. (B) Quantitative data of γH2AX foci in MDA-MB-231 cells transfected with control or *Smyca* ASOs and treated with 50 μM cisplatin for 4 h, followed by drug removal to allow DNA repair for indicated time periods. The knockdown efficiencies of *Smyca* ASOs are shown on the right. (C) Representative images and quantitative data for alkaline comet assay to detect the extent of DNA DSBs in indicated Hs578T derivatives treated with 50 μM cisplatin for 24 h. Bar, 50 μm. Data in all panels are mean ± SD, n = 3 and >30 cells per group per experiment are analyzed. *P* values are determined by one-way ANOVA with Tukey’s post hoc test.

**Fig. S3. FOXM1 mediates the DSB repair-promoting function of *Smyca*.** (A) RIP analysis for the enrichment of *Smyca* in TWIST1 (left panel) or SMARCA4 (right panel) immunoprecipitates derived from MDA‑MB‑231 cells. Data are normalized with that from the control antibody. The presence of TWIST1 or SMARCA4 in the immunoprecipitates are shown on the bottom. Data are mean ± SD, n = 3. *P* values are determined by unpaired t-test. (B) Western blot analysis of FOXM1 in MDA-MB-231 and Hs578T derivatives. (C) Cis regulatory element annotation analysis for FOXM1 ChIP-seq data derived from MDA-MB-231 cells. (D, E) RT-qPCR analysis of the expression of indicated genes in MDA-MB-231 cell derivatives treated with 50 μM cisplatin for 72 h (D), or MDA-MB-231 FOXM1 KO cells transfected with control or *Smyca* ASOs and treated with 50 μM cisplatin for 72 h (E). The KO of FOXM1 is shown on the top panel of (E). (F, G) ChIRP-qPCR assay for *Smyca* occupancy on the loci of indicated FOXM1-targeted genes. GAPDH was used as a control. Tilling biotinylated oligonucleotides complementary to LacZ or *Smyca* were used to pull down the RNA-associated chromatins from MDA-MB-231 cells. Data are normalized with the inputs. Data in (D-G) are mean ± SD, n = 3. *P* values are determined by one-way ANOVA with Tukey’s post hoc test (D, E) or unpaired t-test (F, G). (H) Representative images and quantitative data for alkaline comet assay to detect the extent of DNA DSBs in MDA-MB-231 FOXM1 KO cells transfected with control or *Smyca* ASOs and treated with 50 μM cisplatin for 24 h. Bar, 50 μm. The knockdown efficiencies of *Smyca* ASOs are shown on the bottom right. Data are mean ± SD, n = 3 and >30 cells per group per experiment are analyzed. *P* values are determined by one-way ANOVA with Tukey’s post hoc test.

**Fig. S4. AP-1 promotes the recruitment of *Smyca* and FOXM1 to promoters of the *Smyca*-dependent FOXM1 targets.** (A) The top 4 binding sequences classified by MEME motif analysis for 96 *Smyca*-regulated FOXM1 targets together with the *P* values are shown. (B) ChIRP-qPCR assay for *Smyca* occupancy on the loci of indicated FOXM1-targeted HR genes. Tilling biotinylated oligonucleotides complementary to LacZ or *Smyca* were used to pull down the RNA-associated chromatins from MDA-MB-231 cells treated with or without 20 μM AP-1 inhibitor T5224 (B) for overnight. Data are normalized with the inputs. (C, D) ChIP-qPCR analysis of the recruitment of FOXM1 to the promoters of indicated genes in MDA-MB-231 cells treated with 20 μM T5224 (C) or 5 μM STAT3 inhibitor LLL12 (D) for overnight. The enrichment folds are normalized with that from control antibody (IgG). (E) Cell proliferation of Hs578T or MDA-MB 231 cells stably expressing control shRNA and *Smyca* shRNA. (F) Cell proliferation of MDA-MB 231 cells transiently expressing *Smyca* and treated with 5 μM SB431542, 30 μM FDI-6, and/or 12.5 μM nucleosides for 24 h. The expression levels of *Smyca* are shown on the right. Data in (B), (C), (D), (E), (F) are mean ± SD, n=3. *P* values are determined by unpaired t test (C, D) or one-way ANOVA with Tukey’s post hoc test (B, E, F).

**Fig. S5. *Smyca* depletion sensitizes TNBC cells to cisplatin or olaparib.** (A) Top left panel: The scheme of nude mice orthotopically implanted with indicated MDA-MB-231 cells derivatives and treated with cisplatin or olaparib. Tumor volumes were measured on the indicated days and plotted on the bottom left panel. Tumors were surgically removed on the sacrifice day and their sizes and weights are shown on the middle and right panels, respectively. Data are mean ± SD, n=4. *P* values are determined by two-way ANOVA with Tukey’s post hoc test. (B) IHC analyses with indicated antibodies of the tumor sections derived from Figure 5D (top panel). Bar, 50 μm. The positively stained areas are quantified, normalized to control group, and shown on the bottom panel. Data are mean ± SD, n=4. *P* values are determined by one-way ANOVA with Tukey’s post hoc test. (C) RT-qPCR analysis of indicated genes from tumor samples shown in Figure 5D. Data are mean ± SD, n=3. *P* values are determined by unpaired t test.

**Fig. S6.** ***Smyca* depletion in combined with cisplatin or PARPi treatment activates cGAS/STING pathway and tumor immunogenicity.** (A, B) Micronuclei were stained by DAPI in indicated MDA-MB-231 derivatives treated with 10 μM olaparib (A), or indicated Hs578T derivatives treated with 50 μM cisplatin (B) for 48 h. Bar, 10 μm. Data are mean ± SD, n=3 and >30 cells per group per experiment are analyzed. *P* values are determined by one-way ANOVA with Tukey’s post hoc test. (C) cGAMP levels in indicated Hs578T derivatives treated with 50 μM cisplatin for 72 h. (D, G) Western blot analysis of indicated proteins in Hs578T derivatives treated with 50 μM cisplatin for 72 h. (E) Flag-STING was immunoprecipitated from indicated Hs578T derivatives treated with 50 μM cisplatin for 72 h and transfected with Flag-STING, followed by Western blot analysis with indicated antibodies. (F) RT-qPCR analysis of indicated genes from MDA-MB-231 derivatives treated with 50 μM cisplatin for 72 h. (H, I) Representative confocal images and quantitative data for p65 nuclear localization in indicated MDA-MB-231 (H) or Hs578T (I) derivatives treated with 50 μM cisplatin. Bar, 10 μm. Data are mean ± SD, n=3 and >30 cells per group per experiment are analyzed. *P* values are determined by one-way ANOVA with Tukey’s post hoc test. (J, K) Luciferase assay for IFNβ promoter activity (J) or ELISA assay for secreted IFNβ (K) of indicated MDA-MB-231 derivatives treated with 50 μM cisplatin for 72 h. (L-N) RT-qPCR analysis of indicated genes in Hs578T derivatives treated with 50 μM cisplatin for 72 h (L), or MDA-MB-231 derivatives treated with 50 μM cisplatin for 72 h and 6 μM G140 for 24 h (M), or MDA-MB-231 cells transfected with control or *Smyca* ASO and treated with 10 μM olaparib (N) for 72 h. Data in (C), (F), (J), (K), (L), (M), (N) are mean ± SD, n=3. *P* values are determined by one-way ANOVA with Tukey’s post hoc test. The quantifications in (D), (E), (G) are the intensity ratios of phospho/total protein normalized to loading control.

**Fig. S7. FOXM1 targeting in combined with cisplatin or olaparib treatment activates cGAS-STING pathway.** (A, B) Micronuclei were stained by DAPI in MDA-MB-231 control or FOXM1 KO cells treated with 50 μM cisplatin (A) or 10 μM olaparib (B) for 48 h. Bar, 10 μm. Data are mean ± SD, n=3 and >30 cells per group per experiment are analyzed. *P* values are determined by one-way ANOVA with Tukey’s post hoc test. (C, D) Western blot analysis of indicated proteins in MDA-MB-231 control or FOXM1 KO cells treated with 50 μM cisplatin or (C) or 10 μM olaparib (D) for 72 h. (E-G) RT-qPCR analysis of indicated genes in MDA-MB-231 control or FOXM1 KO cells treated with 50 μM cisplatin (E) or 10 μM olaparib (F) for 72 h, or in 4T1 cells treated with 30 μM FDI-6 for 24 h (G). (H) Luciferase assay of 4T1 cells transfected with Foxm1 reporter and treated with 30 μM FDI-6 for 24 h. Data in (E), (F), (G), (H) are mean ± SD, n=3. *P* values are determined by one-way ANOVA with Tukey’s post hoc test (E, F) or unpaired t-test (G, H). (I, J) Western blot analysis of indicated proteins in 4T1 cells treated with 30 μM FDI-6 for 24 h and 50 μM cisplatin (I) or 10 μM olaparib (J) for 72 h. (K) Tumors surgically removed on the sacrifice day from the experiment in Figure 7C are shown. The quantifications in (C), (D), (I), (J) are the intensity ratios of phospho/total protein normalized to loading control.

**Fig. S8. Gating strategies for indicated TILs.**

**Figure S9. Physicochemical characterization of ASO-loaded LNPs.** (A, B) Representative dynamic light scattering intensity-based size distribution profiles of LNPs loaded with control gapmer ASO (Control LNP) or *Smyca*-targeting gapmer ASO (*Smyca* LNP), respectively. Both formulations showed a single dominant nanoscale population with comparable peak size distributions, indicating that replacement of the control ASO with *Smyca* ASO did not substantially alter particle formation or size homogeneity. (C) Quantification of hydrodynamic particle diameter showed that control LNP and *Smyca* LNP had similar average sizes, consistent with the representative DLS profiles. (D) Polydispersity index (PDI) analysis demonstrated comparable and relatively narrow size distributions between the two LNP formulations. (E) ζ-potential measurements showed similar surface charge profiles for control LNP and *Smyca* LNP, indicating that ASO sequence substitution did not markedly affect the overall surface electrostatic properties of the particles. (F) Quantification of ASO encapsulation efficiency showed similarly high loading efficiency for both control and *Smyca* ASOs. Together, these data demonstrate that control LNP and *Smyca* LNP possess comparable size, dispersity, surface charge, and ASO-loading characteristics, supporting their use as matched delivery formulations for assessing the biological effects of *Smyca* ASO. Data are presented as mean ± SD from three independent preparations or measurements.

**Table S1:** IPA upstream regulator analysis for identifying common upstream regulators of *Smyca*-upregulated genes

| **Upstream Regulators** | **Z score** | ***P* value** |
| --- | --- | --- |
| MYC | 4.625 | 2.55E-14 |
| SMARCA4 | 3.896 | 1.61E-15 |
| TP63 | 3.356 | 2.81E-09 |
| CREB1 | 2.91 | 0.00531 |
| HIF1A | 2.518 | 3.438E-10 |
| SOX11 | 2.419 | 0.000091 |
| SREBF1 | 2.331 | 0.0000626 |
| MED1 | 2.313 | 0.000825 |
| MITF | 2.273 | 0.000091 |
| STAT3 | 2.27 | 0.0097 |
| SMAD3 | 2.263 | 0.0080132 |
| CTNNB1 | 2.233 | 0.0054221 |
| CNOT7 | 2.229 | 0.000000973 |
| TWIST2 | 2.215 | 0.0125 |
| E2F1 | 2.201 | 0.00326 |
| ERG | 2.182 | 0.00026 |
| FOXC2 | 2.096 | 0.0155 |
| FOXM1 | 2.073 | 0.0095 |
| WT1 | 1.89 | 0.000133 |
| MYCN | 1.872 | 0.012 |
| HNF1A | 1.863 | 0.023 |
| POU2F2 | 1.852 | 0.0092 |
| TCF7L2 | 1.793 | 0.0073 |
| TWIST1 | 1.727 | 0.023 |
| SP1 | 1.69 | 0.00000022 |

**Table S2:** The *Smyca*-regulated FOXM1 target genes identified by intersecting the ChIP-seq with RNA-seq data

| **Gene Symbol** | **Gene ID** | **ChIP-seq**  **(Fold change: sh*Smyca*/**  **shCtrl)** | **RNA-seq**  **(Fold change: sh*Smyca*/**  **shCtrl)** | ***P* value** |
| --- | --- | --- | --- | --- |
| BRCA2 | ENSG00000139618 | 0.3822 | 0.32603 | 2.41E-06 |
| RAD51 | ENSG00000051180 | 0.4034 | 0.41592 | 2.83E-27 |
| NBS1 | ENSG00000104320 | 0.4381 | 0.38757 | 2.5E-07 |
| BRIP | ENSG00000136492 | 0.4315 | 0.3513 | 0.003653 |
| POLE2 | ENSG00000100479 | 0.4096 | 0.42577 | 3.41E-19 |
| EXO1 | ENSG00000174371 | 0.3522 | 0.43145 | 3.22E-06 |
| RFC2 | ENSG00000049541 | 0.4961 | 0.38804 | 1.31E-05 |
| MCM4 | ENSG00000104738 | 0.3631 | 0.41258 | 7.21E-09 |
| POLE | ENSG00000177084 | 0.4964 | 0.49036 | 3.83E-07 |
| BARD1 | ENSG00000138376 | 0.4072 | 0.36736 | 4.43E-32 |
| CLSPN | ENSG00000092853 | 0.4262 | 0.36913 | 0.000313 |
| MDC1 | ENSG00000137337 | 0.3065 | 0.4268 | 0.002399 |
| ASPM | ENSG00000066279 | 0.43814 | 0.3912 | 1.14E-07 |
| KIF2C | ENSG00000142945 | 0.37956 | 0.35476 | 0.002401 |
| POLD | ENSG00000106628 | 0.3536 | 0.40059 | 5.28E-17 |
| RPA3 | ENSG00000106399 | 0.28021 | 0.4207 | 6.07E-10 |
| FEN1 | ENSG00000168496 | 0.3181 | 0.39746 | 1.97E-13 |
| RRM1 | ENSG00000167325 | 0.49248 | 0.47772 | 8.75E-38 |
| RRM2 | ENSG00000171848 | 0.37588 | 0.42162 | 1.26E-25 |
| AK1 | ENSG00000106992 | 0.32247 | 0.33261 | 0.000155 |
| DTYMK | ENSG00000168393 | 0.33818 | 0.35363 | 4.55E-10 |
| NEM1 | ENSG00000175826 | 0.29733 | 0.42971 | 1.34E-15 |
| CDKN2B | ENSG00000147883 | 0.36528 | 0.47227 | 1.42E-07 |
| CDKN1A | ENSG00000124762 | 0.37404 | 0.30281 | 5.34E-26 |
| PA2G4 | ENSG00000170515 | 0.37221 | 0.41442 | 4.7E-09 |
| FAM64A | ENSG00000129195 | 0.42194 | 0.48058 | 0.002214 |
| HMMR | ENSG00000072571 | 0.35387 | 0.3662 | 5.4E-150 |
| PTTG1 | ENSG00000164611 | 0.49738 | 0.35584 | 2.68E-27 |
| FZR1 | ENSG00000105325 | 0.32052 | 0.32406 | 5.06E-18 |
| MZT1 | ENSG00000204899 | 0.36094 | 0.41455 | 0.003265 |
| MALAT1 | ENSG00000251562 | 0.41119 | 0.30542 | 3.92E-06 |
| AMOTL2 | ENSG00000114019 | 0.48635 | 0.47699 | 0.000328 |
| C21orf58 | ENSG00000160298 | 0.47137 | 0.29898 | 4.41E-28 |
| EEF1A1 | ENSG00000156508 | 0.4601 | 0.41987 | 0.000763 |
| GPSM2 | ENSG00000121957 | 0.36507 | 0.3587 | 9.58E-07 |
| NUF2 | ENSG00000143228 | 0.416 | 0.35456 | 0.00084 |
| LBR | ENSG00000143815 | 0.41252 | 0.40775 | 1.28E-50 |
| PRR11 | ENSG00000068489 | 0.39914 | 0.33505 | 3.49E-20 |
| TUBA1C | ENSG00000167553 | 0.44088 | 0.41577 | 8.3E-05 |
| H2AFX | ENSG00000188486 | 0.44603 | 0.41596 | 2.62E-05 |
| KNSTRN | ENSG00000128944 | 0.44304 | 0.42128 | 0.000126 |
| LOC100127888 | ENSG00000232803 | 0.41393 | 0.42988 | 9.85E-21 |
| LOC284801 | ENSG00000227195 | 0.40888 | 0.3357 | 4.32E-08 |
| MIR22HG | ENSG00000186594 | 0.34609 | 0.39224 | 1.31E-11 |
| MIR3687 | ENSG00000198982 | 0.45334 | 0.47427 | 2.39E-05 |
| MIR4461 | ENSG00000263963 | 0.35742 | 0.41692 | 1.61E-19 |
| ASXL1 | ENSG00000171456 | 0.37956 | 0.35992 | 2.17E-05 |
| DYNLL1 | ENSG00000088986 | 0.41961 | 0.3671 | 8.26E-07 |
| CD68 | ENSG00000129226 | 0.4969 | 0.48797 | 1.75E-42 |
| PCBP1 | ENSG00000169564 | 0.34262 | 0.36889 | 0.000166 |
| ZNF335 | ENSG00000198026 | 0.44225 | 0.31169 | 4.4E-05 |
| TPX2 | ENSG00000088325 | 0.41457 | 0.28477 | 5.73E-22 |
| DCLRE1C | ENSG00000152457 | 0.42028 | 0.31714 | 2.6E-12 |
| LINC00273 | ENSG00000256642 | 0.34085 | 0.34455 | 1.45E-12 |
| MT2A | ENSG00000149480 | 0.32603 | 0.41006 | 1.08E-11 |
| MIRLET7BHG | ENSG00000197182 | 0.41592 | 0.37803 | 0.000262 |
| MPDU1 | ENSG00000129255 | 0.38757 | 0.36013 | 5.14E-19 |
| MRPS18B | ENSG00000204568 | 0.3513 | 0.30255 | 4.59E-33 |
| CEP192 | ENSG00000101639 | 0.41457 | 0.40357 | 0.001056 |
| CKAP2 | ENSG00000136108 | 0.41455 | 0.34168 | 0.000203 |
| FAM83D | ENSG00000101447 | 0.41442 | 0.42106 | 0.000238 |
| NEURL1B | ENSG00000214357 | 0.41437 | 0.40458 | 0.000367 |
| MKI67 | ENSG00000148773 | 0.41409 | 0.33243 | 1.2E-28 |
| ARL6IP1 | ENSG00000170540 | 0.41403 | 0.45614 | 5.43E-09 |
| HIST1H3B | ENSG00000286522 | 0.41393 | 0.35673 | 0.001625 |
| KRI1 | ENSG00000129347 | 0.4132 | 0.30657 | 0.00017 |
| SNHG17 | ENSG00000196756 | 0.41258 | 0.45904 | 9.39E-09 |
| SNORD12C | ENSG00000209042 | 0.41252 | 0.45918 | 0.001197 |
| SOGA1 | ENSG00000214338 | 0.4115 | 0.30473 | 3.92E-45 |
| STX16 | ENSG00000124222 | 0.41119 | 0.32693 | 0.000563 |
| REEP4 | ENSG00000168476 | 0.41103 | 0.48578 | 2.08E-49 |
| RGS5 | ENSG00000143248 | 0.41041 | 0.36124 | 3.64E-08 |
| SCAND1 | ENSG00000171222 | 0.41006 | 0.36659 | 2.5E-12 |
| TUBA1C | ENSG00000167553 | 0.40964 | 0.37557 | 0.000338 |
| TUBB4B | ENSG00000188229 | 0.40949 | 0.35575 | 3.05E-16 |
| UBALD2 | ENSG00000185262 | 0.40888 | 0.35893 | 1.09E-20 |
| UBE2C | ENSG00000175063 | 0.4085 | 0.37443 | 1.36E-08 |
| UBE2S | ENSG00000108106 | 0.40825 | 0.26854 | 2.44E-07 |
| LRRC14 | ENSG00000106959 | 0.40786 | 0.49623 | 8.28E-07 |
| PFN1 | ENSG00000108518 | 0.40775 | 0.47479 | 5.55E-16 |
| PGAP2 | ENSG00000148985 | 0.40761 | 0.42211 | 0.00211 |
| PHF19 | ENSG00000119403 | 0.40664 | 0.3051 | 5.93E-57 |
| WDR81 | ENSG00000276021 | 0.40585 | 0.27829 | 6.61E-05 |
| MFSD3 | ENSG00000167700 | 0.40478 | 0.31981 | 6.85E-18 |
| RNF167 | ENSG00000108523 | 0.40458 | 0.32062 | 3.4E-07 |
| RNF26 | ENSG00000173456 | 0.40442 | 0.38464 | 1.23E-38 |
| NCAPD2 | ENSG00000010292 | 0.40434 | 0.39531 | 1.05E-24 |
| HP1BP3 | ENSG00000127483 | 0.40393 | 0.38005 | 2.23E-13 |
| RCCD1 | ENSG00000166965 | 0.40367 | 0.3697 | 6.36E-13 |
| RECQL4 | ENSG00000160957 | 0.40357 | 0.42464 | 1.45E-09 |
| SLC25A11 | ENSG00000108528 | 0.40282 | 0.41437 | 1.75E-06 |
| PTMA | ENSG00000187514 | 0.40138 | 0.38856 | 2.13E-20 |
| PIM3 | ENSG00000198355 | 0.40119 | 0.32836 | 2.5E-27 |
| IFT80 | ENSG00000068885 | 0.40116 | 0.33308 | 9.53E-21 |
| LGALS1 | ENSG00000100097 | 0.40067 | 0.39162 | 1.28E-12 |
| GTSE1 | ENSG00000075218 | 0.40059 | 0.40393 | 1.01E-06 |

**Table S3:** The functions of *Smyca*- and/or FOXM1- regulated target genes described in this study

| **Genes** | ***Smyca* Target** | **FOXM1 Target** | **Function** |
| --- | --- | --- | --- |
| BRCA2 | Yes | Yes^1^ | HR |
| RAD51 | Yes | Yes^1^ | HR |
| NBS1 | Yes | Yes^1^ | HR |
| BRIP | Yes | Yes^1^ | HR |
| POLE2 | Yes | Yes^1^ | HR |
| EXO1 | Yes | Yes^1^ | HR |
| RFC2 | Yes | Yes^1^ | HR |
| XRCC1 | No | Yes^1^ | NHEJ |
| PLK1 | No | Yes^1^ | Cell cycle regulation |
| CCNB1 | No | Yes^1^ | Cell cycle regulation |
| CENP | No | Yes^1^ | Cell cycle regulation |
| AURKB | No | Yes^1^ | Cell cycle regulation |
| CT45A1 | Yes | Yes^2^ | Cancer antigen |
| FAM133A | Yes | Yes^2^ | Cancer antigen |
| ARX | Yes | Yes^2^ | Cancer antigen |
| TMEFF1 | Yes | Yes^2^ | Cancer antigen |
| B2M | Yes | Yes^2^ | Antigen presenting gene |
| BAP2 | Yes | Yes^2^ | Antigen presenting gene |
| RRM1 | Yes | Yes^1^ | dNTP synthesis |
| RRM2 | Yes | Yes^1^ | dNTP synthesis |
| DTYMK | Yes | Yes^1^ | dNTP synthesis |

^1^: FOXM1 targets identified by this study.

^2^: FOXM1 targets identified by *Nature* **2025**, *638* (8052), 1112 ^[1]^

**Table S4:** Sequences and sources of shRNAs, sgRNAs, ASOs, and ChIRP probes

| shRNA | sequence( 5’ to 3’) | source (identifiers) |
| --- | --- | --- |
| sh*control* | CAACAAGATGAAGAGCACCAAA | RNA Technology Platforms and Gene Manipulation Core Facility, Taipei, Taiwan |
| sh*Smyca* #1 | GCATAGATAGGTGGGTGAGTG | BLOCK-iT™ RNAi  Designer |
| sh*Smyca* #2 | GCTGATGCTTGGAGCAGAGAT | BLOCK-iT™ RNAi  Designer |
| sh*control*  (Hs578T) | CTTACGCTGAGTACTTCGAGTG | RNA Technology Platforms and Gene Manipulation Core Facility, Taipei, Taiwan |
| sh*Smyca* #1  (Hs578T) | GGGCATGGAACAAGTTCCTTGTG | BLOCK-iT™ RNAi  Designer |
| sh*Smyca* #2  (Hs578T) | GGCATGGAACAAGTTCCTTGT | BLOCK-iT™ RNAi  Designer |

| sgRNA | sequence (5’ to 3’) | source (identifiers) |
| --- | --- | --- |
| sgFOXM1 #1 (exon1) | TGAGAATCAGTGGCCGACGG | RNA Technology Platforms and Gene Manipulation Core Facility, Taipei, Taiwan |
| sgFOXM1 #2  (exon1) | CATGCCCAACACGCAAGTAG | RNA Technology Platforms and Gene Manipulation Core Facility, Taipei, Taiwan |
| sgBRCA1  (exon9) | CACCCAGTTTGGATTCTGCAAAA | constructed in-house |

| ASO | sequence (5’ to 3’) | source (identifiers) |
| --- | --- | --- |
| Control ASO | GCTCCCTTCAATCCAA | Qiagen |
| *Smyca* ASO #1 | CGCATTGGGAGACTAA | Qiagen |
| *Smyca* ASO #2 | GAGTTGGCATTTAACG | Qiagen |

| ChIRP Probe name | | sequence (5’ to 3’) |
| --- | --- | --- |
| *LacZ* | ChIRP *LacZ-*1 | TAGCCAGCTTTCATCAACAT |
|  | ChIRP *LacZ-*2 | AGCAGCAGACCATTTTCAAT |
|  | ChIRP *LacZ-*3 | GTGTGGGCCATAATTCAATT |
|  | ChIRP *LacZ-*4 | CGGCAGCCGTTATTATTATT |
|  | ChIRP *LacZ-*5 | GAAACTGTTACCCGTAGGTA |
|  | ChIRP *LacZ-*6 | CACGGCGTTAAAGTTGTTCT |
|  | ChIRP *LacZ-*7 | GGATCGACAGATTTGATCCA |
|  | ChIRP *LacZ-*8 | GTAGTTCAGGCAGTTCAATC |
|  | ChIRP *LacZ-*9 | CAACGGTAATCGCCATTTGA |
|  | ChIRP *LacZ-*10 | TGCAAGGCGATTAAGTTGGG |
| *Smyca* | ChIRP *Smyca-*1 | CTCTGCTCCAAGCATCAG |
|  | ChIRP *Smyca-*2 | CCTGCAGCACACATTTGG |
|  | ChIRP *Smyca-*3 | CTCACCCACCTATCTATG |
|  | ChIRP *Smyca-*4 | CAGGCTTGGGAGAGGAGG |
|  | ChIRP *Smyca-*5 | TTTAACGGTCTGGAGGCC |
|  | ChIRP *Smyca-*6 | GTTAGAAAGCTGCAGGGC |
|  | ChIRP *Smyca-*7 | CTGCTTGTGTCCCTTCTG |
|  | ChIRP *Smyca-*8 | AGAGTCCTAACTCTTCCC |
|  | ChIRP *Smyca-*9 | TCAGTCCCACCCAGAAAA |
|  | ChIRP *Smyca-*10 | CTTCTGTCCACGACTACC |
|  | ChIRP *Smyca-*11 | GAAGTCAGGGCAACTTTT |

**Table S5:** Sequences of qPCR primers, ChIRP primers, RCA template and primers, and primers for generating FOXM1 binding element

| qPCR primer | | |
| --- | --- | --- |
| Gene name | | Sequence (5’ to 3’) |
| GAPDH | F | TGTTGCCATCAATGACCCCTT |
|  | R | CTCCACGACGTACTCAGCG |
| *Smyca* | F | TTGAATTCCTGGGCACAAGT |
|  | R | CACACCTGAGCCACCTGTAA |
| NBS1 | F | TCTGTCAGGACGGCAGGAAAGA |
|  | R | CACCTCCAAAGACAACTGCGGA |
| EXO1 | F | TCGGATCTCCTAGCTTTTGGCTG |
|  | R | AGCTGTCTGCACATTCCTAGCC |
| BRIP | F | GGAAACAGTCAAGAGTCATCGAA |
|  | R | TCTGAGCAATCTGCTTGTGTG |
| BRCA2 | F | ACAAGCAACCCAAGTGTCAAT |
|  | R | TGAAGCTACCTCCAAAACTGTG |
| RAD51 | F | CAACCCATTTCACGGTTAGAGC |
|  | R | TTCTTTGGCGCATAGGCAACA |
| POLE2 | F | TGCGTCCGTTTTCCTAGCAGCA |
|  | R | GGGCAGACATAAAGAGGTAGGG |
| RFC2 | F | GTCGGGAATGAAGACACCGTGA |
|  | R | CAGAATGCTTGTGGTCTTGCCG |
| XRCC1 | F | CCTTTGGCTTGAGTTTTGTACG |
|  | R | CCTCCTTCACACGGAACTGG |
| RRM1 | F | GCCAGGATCGCTGTCTCTAAC |
|  | R | GAGAGTGTTTGCCATTATGTGGA |
| RRM2 | F | CACGGAGCCGAAAACTAAAGC |
|  | R | TCTGCCTTCTTATACATCTGCCA |
| DTYMK | F | CCGGTTCCCGGAAAGATCAAC |
|  | R | TCCCAGCGATTTGCAGAAAAA |
| FOXM1 | F | CGTCGGCCACTGATTCTCAAA |
|  | R | GGCAGGGGATCTCTTAGGTTC |
| BRCA1 | F | GAATAGAAACTACCCATCTCAAGAGGA |
|  | R | CAGGTAAGGGGTTCCCTCTAGAT |
| MRE11 | F | GGGGCAGATGCACTTTGTG |
|  | R | GAAGCAAAACCGGACTAATGTCT |
| RAD50 | F | TACTGGAGATTTCCCTCCTGG |
|  | R | AGACTGACCTTTTCACCATGC |
| RPA2 | F | GGTAGCCTTTAAGATCATGCCC |
|  | R | CTGTTGGCTTTGCTTAGTACCA |
| IFNA1 | F | AGAAGGCTCCAGCCATCTCTGT |
|  | R | TGCTGGTAGAGTTCGGTGCAGA |
| IFNA2 | F | TGGGCTGTGATCTGCCTCAAAC |
|  | R | CAGCCTTTTGGAACTGGTTGCC |
| IFNA8 | F | GTTCCAGAAGGCTCAAGCCATC |
|  | R | GAAGGGTCTCATCCAAAGCAGC |
| IFNA10 | F | GTTCCAGAAGGCTCAAGCCATC |
|  | R | TAGGAGGCTCTGTTCCCAAGCA |
| IFNA14 | F | TCCTGCCTGAAGGACAGACATG |
|  | R | GCTGCATCATCTCATGGAGGAC |
| IFNA16 | F | ATCTGCCTCAGACTCACAGCCT |
|  | R | CTGGTTGCCATCAAACACCTCC |
| IFNA21 | F | GTTCCAGAAGGCTCAAGCCATC |
|  | R | GAGGCTCTGTTCCCAAGTAGCA |
| IFNB | F | GTCACTGTGCCTGGACCATAG |
|  | R | GTTTCGGAGGTAACCTGTAAGTC |
| CXCL10 | F | GTGGCATTCAAGGAGTACCTC |
|  | R | TGATGGCCTTCGATTCTGGATT |
| cGAS | F | AGGAAGCAACTACGACTAAAGCC |
|  | R | CGATGTGAGAGAAGGATAGCCG |
| STING | F | CCTGAGTCTCAGAACAACTGCC |
|  | R | GGTCTTCAAGCTGCCCACAGTA |
| USP18 | F | CCTGAGGCAAATCTGTCAGTC |
|  | R | CGAACACCTGAATCAAGGAGTTA |
| IFIT3 | F | TCAGAAGTCTAGTCACTTGGGG |
|  | R | CGAACACCTGAATCAAGGAGTTA |
| CT45A1 | F | GCACCTGTGGGAGGAAACG |
|  | R | CCTGACTGCAGTAGGTCCTTG |
| FAM133A | F | TGGAGAGGCCCAACTCAATCT |
|  | R | GGTGGGTCTTGGTCGATTTAFA |
| ARX | F | CTGCTGAAACGCAAACAGAGGC |
|  | R | CTCGGTCAAGTCCAGCCTCATG |
| TMEFF1 | F | TGCCCTGAAAACCTCAATGGT |
|  | R | CCAGTGTAGCCAGATTCACATC |
| B2M | F | AGACCTTTGGGCTGCCTTAT |
|  | R | TAGCCTCCCTCACTCCAAGA |
| TAP2 | F | GACCTTCCACTAGACCATGAGC |
|  | R | GGAGGATTAAGATTAGTACGATGGT |
| Nbs1  (mouse) | F | CAGACCTATTCCTGAAGCGGAG |
|  | R | AAGGCTTGGTCCTGGAGTCGTT |
| Brca2  (mouse) | F | GAGCACAGATGGAGACAGGGAA |
|  | R | CAGACGGAGATGTCGCCTTTCT |
| Brip  (mouse) | F | CAGTAGCAAGCCACGGTGTTTC |
|  | R | GCCAGTTGACTGGCTCTGTTTG |
| Rad51  (mouse) | F | TGGAGGCTGTTGCTTATGCACC |
|  | R | GCTGGTGAAACTCAGTTGCCGT |

| ChIRP-qPCR and ChIP-qPCR primer | | |
| --- | --- | --- |
| Gene name | | Sequence (5’ to 3’) |
| NBS1  (promoter) | F | AATTAAAAATTTTCCTTATGTTGCTTT |
|  | R | GGGCGCTTGCCCGCCACCTGGTGGTTGG |
| EXO1  (promoter) | F | TGCCATCATGATTTGTGACTTAACTGTCT |
|  | R | GTTCCAGGTGGCATGGAGGCA |
| BRIP  (promoter) | F | AGACTCTATCGCCGGTTTCA |
|  | R | AAACAAGGGCTCAAGGTACG |
| BRCA2  (promoter) | F | CATAAGGGGGCAGAATAAGAGTTG |
|  | R | TGATAGAAGGTGGAAATGAGGTCC |
| RAD51  (promoter) | F | AGGTTTCACCACGTTGGCCAGG |
|  | R | AAAGGCTGAGTAGGGCCGGG |
| POLE2  (promoter) | F | CCGAGAGTGGGCCCGAGACA |
|  | R | CATGCGCGGCAGACCTTCCT |
| RFC2  (promoter) | F | GTGGTCTTTGTCCATTTTGGCTGC |
|  | R | GGAGTGAACCCTCACCAGCCACT |
| RRM1  (promoter) | F | ATAGGTGAAGGCGGCTGT |
|  | R | TAAAGGTATGGCAGGGAGTGG |
| RRM2  (promoter) | F | TGGCGATGAATGGCGAGTTG |
|  | R | TCCCCACCTAGGTCTGTTTC |
| DTYMK  (promoter) | F | CACGCCCTCCAGCACTATG |
|  | R | CGAGGCCGGGAAATACTAGC |
| PLK1  (promoter) | F | CCAGAGGGAGAAGATGTCCA |
|  | R | GTCGTTGTCCTCGAAAAAGC |
| AURKB  (promoter) | F | GGGGTCCAAGGCACTGCTAC |
|  | R | GGGGCGGGAGATTGAAAAG |
| CCNB1  (promoter) | F | CGCGATCGCCCTGGAAACGCA |
|  | R | CCCAGCAGAAACCAACAGCCGT |
| CCNB1  (promoter) | F | CGCGATCGCCCTGGAAACGCA |
|  | R | CCCAGCAGAAACCAACAGCCGT |
| CENP  (promoter) | F | CACCTCCAGTAGAGGGGCTTG |
|  | R | TACCTCCACGCCTATTGGTC |
| GAPDH  (promoter) | F | AGCTCAGGCCTCAA GACCTT |
|  | R | AAGAAGATGCGGCTGACTGT |

| RCA template, probe, and primers | sequence (5’ to 3’) |
| --- | --- |
| primer on template | CTCTGGTGTGAGTCCCAAGTCCTGGACTAT |
| Template probe | PO_4_-GGACTCACACCAGACGATGATTCTGTGATCCAAC  GTCTTCGAGATTGTGATGACATAGTCCAGGACTTG  -OH |
| qPCR primer | CTCTGGTGTGAGTCCCAAG |
|  | CACACCAGAGGATGATTCTG |

| FOXM1 reporter construct | | Sequence (5’ to 3’) |
| --- | --- | --- |
| 6X FOXM1  binding element  (FKH motif) | F | CTAGCTTTGTTTATTTGTTTGTTTATTTGTTTGTTTATTTGA |
|  | R | AGCTTCAAATAAACAAACAAATAAACAAACAAATAAACAAAG |

| FOXM1 deletion mutant constructs | | Sequence (5’ to 3’) |
| --- | --- | --- |
| FOXM1 dNRD | F | CTATCGATTGAATTCATGCGGCCACCCTACTCT |
|  | R | AGAGTAGGGTGGCCGCATGAATTCAATCGATAG |
| FOXM1 dDBD | F | AACTCTGTGTCTGAGAGTGCCAACCGCTAC |
|  | R | GTAGCGGTTGGCACTCTCAGACACAGAGTT |
| FOXM1 dTAD | F | GGAGGACCTTTTAAGGGATCCTCTAGAGTC |
|  | R | GACTCTAGAGGATCCCTTAAAAGGTCCTCC |
| FOXM1 dIDR-TAD | F | CCGGAATTCATGAAAACTAGCCCCCGTCGG |
|  | R | CGCGGATCCGGGGTGAATGGTCCAGAAGG |

**Table S6:** Antibody list

| Protein | Vendor | Cat. number | Source | Titer |
| --- | --- | --- | --- | --- |
| GAPDH | GeneTex | GTX100118 | Rabbit | WB (1:10000) |
| HA | Cell Signaling | 3724S | Rabbit | WB (1:1000) |
| FOXM1 | GeneTex | GTX102170 | Rabbit | IP (1:500)  ChIP (1:200) |
| FOXM1 [C3], C-term | GeneTex | GTX100276 | Rabbit | WB (1:1000) |
| TWIST1 | GeneTex | GTX60776 | Mouse | WB (1:1000) |
| SMARCA4  (BRG1) | Abcam | ab108318 | Rabbit | WB (1:1000) |
| Rabbit IgG | Abcam | ab171870 | Rabbit | IP (1:100) |
| EasyBlot Rabbit IgG | GeneTex | GTX221666-01 | Rabbit | ChIP (1:1000) |
| TBK1 | Cell Signaling | 3504 | Rabbit | WB (1:1000) |
| IRF3 | Cell Signaling | 11904 | Rabbit | WB (1:1000) |
| IKKβ | Cell Signaling | 2684 | Rabbit | WB (1:1000) |
| p65 | Cell Signaling | 8242 | Rabbit | WB (1:1000)  IF (1:250) |
| IκBα | Cell Signaling | 9242 | Rabbit | WB (1:1000) |
| p-TBK1 | GeneTex | GTX02815 | Rabbit | WB (1:1000) |
| p-IRF3 | GeneTex | GTX02883 | Rabbit | WB (1:1000) |
| p-IKKβ | Cell Signaling | 2697 | Rabbit | WB (1:1000) |
| p-p65 | Cell Signaling | 3033 | Rabbit | WB (1:1000) |
| p-STING | Cell Signaling | 19781 | Rabbit | WB (1:1000) |
| Tubulin | GeneTex | GTX112141 | Rabbit | WB (1:3000) |
| Flag | GeneTex | GTX115043 | Rabbit | WB (1:5000) |
| Rabbit IgG HRP | GE Healthcare | NA934 | Donkey | WB (1:5000) |
| Mouse IgG HRP | GE Healthcare | NA931 | Sheep | WB (1:5000) |
| RAD51 | Santa Cruz | sc-398587 | Mouse | IF (1:500) |
| BRCA1 | Santa Cruz | sc-6954 | Mouse | IF (1:500) |
| p-histone H2AX | Cell Signaling | 9718s | Rabbit | IF (1:500)  IHC (1:500) |
| Goat anti-rabbit  (Alexa 488) | Invitrogen | A-11008 | Goat | IF (1:500) |
| BrdU | Abcam | Ab6326 | Rat | DNA fiber assay  (1:50) |
| BrdU | BD Biosciences | 347580 | Mouse | DNA fiber assay  (1:100) |
| Goat anti-rat  (Alexa 594) | Invitrogen | A-11007 | Goat | DNA fiber assay  (1:100) |
| Goat anti-mouse  (Alexa 488) | Invitrogen | A-11001 | Goat | DNA fiber assay  (1:100) |
| Ki67 | Abcam | ab16667 | Rabbit | IHC (1:1000) |
| Cleaved caspase-3 | Cell Signaling | 9664 | Rabbit | IHC (1:1000) |
| iNOS (M1) | Abcam | ab115819 | Rabbit | IHC (1:500) |
| Mannose receptor (M2) | Abcam | ab64693 | Rabbit | IHC (1:500) |
| CD45  (Pacific blue) | BioLegend | 103126 | Rat | Flow (0.25μg/test) |
| CD3ε (PerCP/Cy5.5) | BioLegend | 100328 | Armenian Hamster | Flow (2μg/test) |
| CD4 (BV785) | BioLegend | 100552 | Rat | Flow (0.25μg/test) |
| CD8 (BV510) | BioLegend | 100752 | Rat | Flow (1μg/test) |
| Pd-1 (Alexa700) | R&D systems | 1712861 | Rat | Flow (0.4μg/test) |
| Foxp3 (Alexa647) | BioLegend | 126408 | Rat | Flow (1.5μg/test) |
| Granzyme B (PE/Cy7) | BioLegend | 372214 | Mouse | Flow (5μg/test) |
| CD11b (Alexa594) | BioLegend | 101254 | Rat | Flow (0.25μg/test) |
| F4/80 (BV605) | BioLegend | 123133 | Rat | Flow (0.5μg/test) |
| IA-IE (FITC) | BioLegend | 107606 | Rat | Flow (0.25μg/test) |
| CD206 (PE) | BioLegend | 141706 | Rat | Flow (0.25μg/test) |

**Reference**

1.	Xie, F., Zhou, X., Ran, Y., et al., “Targeting FOXM1 condensates reduces breast tumour growth and metastasis.” *Nature* **2025**. 638(8052):1112, [*https://doi.org/10.1038/s41586-024-08421-w*](https://doi.org/10.1038/s41586-024-08421-w)*.*
